# Design, Implementation and Practical Evaluation of an IoT Home Automation System for Fog Computing Applications Based on MQTT and ZigBee-WiFi Sensor Nodes

**DOI:** 10.3390/s18082660

**Published:** 2018-08-13

**Authors:** Iván Froiz-Míguez, Tiago M. Fernández-Caramés, Paula Fraga-Lamas, Luis Castedo

**Affiliations:** Department of Computer Engineering, Faculty of Computer Science, Universidade da Coruña, 15071 A Coruña, Spain; ivan.froiz@udc.es (I.F.-M.); luis@udc.es (L.C.)

**Keywords:** IoT, home automation, MQTT, WSN, wireless sensor networks, ZigBee, WiFi, HAS, fog computing

## Abstract

In recent years, the improvement of wireless protocols, the development of cloud services and the lower cost of hardware have started a new era for smart homes. One such enabling technologies is fog computing, which extends cloud computing to the edge of a network allowing for developing novel Internet of Things (IoT) applications and services. Under the IoT fog computing paradigm, IoT gateways are usually utilized to exchange messages with IoT nodes and a cloud. WiFi and ZigBee stand out as preferred communication technologies for smart homes. WiFi has become very popular, but it has a limited application due to its high energy consumption and the lack of standard mesh networking capabilities for low-power devices. For such reasons, ZigBee was selected by many manufacturers for developing wireless home automation devices. As a consequence, these technologies may coexist in the 2.4 GHz band, which leads to collisions, lower speed rates and increased communications latencies. This article presents ZiWi, a distributed fog computing Home Automation System (HAS) that allows for carrying out seamless communications among ZigBee and WiFi devices. This approach diverges from traditional home automation systems, which often rely on expensive central controllers. In addition, to ease the platform’s building process, whenever possible, the system makes use of open-source software (all the code of the nodes is available on GitHub) and Commercial Off-The-Shelf (COTS) hardware. The initial results, which were obtained in a number of representative home scenarios, show that the developed fog services respond several times faster than the evaluated cloud services, and that cross-interference has to be taken seriously to prevent collisions. In addition, the current consumption of ZiWi’s nodes was measured, showing the impact of encryption mechanisms.

## 1. Introduction

The Internet of Things (IoT) paradigm proposes the interconnection of physical devices through networks that allow for sharing data and for controlling their capabilities in real time. It is easy to observe that such a paradigm has a direct application to home automation, a field that integrates automation, computer science and new communication technologies, all aimed at improving comfort, safety and, ultimately, well-being within our homes. Such a field has progressed remarkably in the last decade, introducing new technologies like Augmented Reality (AR) [1,2], and evolving from systems composed by passive objects that react according to the user’s input, to systems based on autonomous sources of information that interact with the environment and anticipate and predict the actions of the home residents.

One of the technologies that has contributed most to the progress of home automation is cloud computing, which offloads home devices from computational-intensive tasks. Nonetheless, in certain home automation scenarios where a fast response and low communications overhead are required, other paradigms have been successful by moving the computing capabilities from the cloud towards the edge of the network [3]. One such paradigm is fog computing, which moves the cloud computational and communication capabilities close to the sensor nodes in order to minimize latency, to distribute computational and storing resources, to enhance mobility and location awareness, and to ease network scalability while providing connectivity among devices in different physical environments [4,5].

To provide such benefits for home automation, four elements have to interact: IoT nodes that collect data (sensor nodes), IoT nodes that embed actuators (actuator nodes), the cloud, and interconnected IoT gateways that exchange messages with the IoT nodes and with the cloud. Communications can be performed through different technologies that, in the case of home automation, are aimed at improving energy efficiency, safety, comfort, and providing audio/video/control systems. These technologies have been proposed for communicating the sensors and actuators installed in a home, either by using independent wired infrastructure (e.g., KNX [6] or LonWorks [7]) or the already existent infrastructure (e.g., X10 [8]). Among these technologies, some of the latest make use of Wireless Sensor Networks (WSNs), which have been applied successfully to fields like train monitoring [9], telemetry [10,11], Industry 4.0 [12,13,14,15] or public safety [16,17]. As of writing, ZigBee is arguably the most popular technology for creating WSNs and it has been included in some of the latest commercial [18] and academic home automation developments [19,20,21,22,23,24,25].

In addition, WiFi networks (i.e., networks based on the IEEE 802.11 family of standards) have become widespread throughout the world and are one of the most popular ways for accessing the Internet due to their flexibility and low deployment cost. However, although the use of commercial WiFi home automation devices is not as popular as the use of ZigBee-based systems, the growing popularity of the IoT paradigm has led to new applications that base their data communications on WiFi networks [26,27,28].

This article presents ZiWi, a fog computing Home Automation System (HAS) that bridges the gap between ZigBee and WiFi devices by connecting sensors and actuators seamlessly to make use of such technologies in a home. ZiWi uses WiFi for actuator nodes since, in general, they have to be continuously awake, listening for asynchronous commands, while ZigBee is implemented by the sensor nodes because it is ideal for sending data at periodic intervals in order to save power (since many nodes rely on batteries). Moreover, the system focuses on the growing IoT market and on easing the connection of emerging sensor technologies. Furthermore, ZiWi makes use of the fog computing paradigm to provide connectivity between the user and the different home appliances, not only allowing the user to control them, but also offering automatisms to simplify tasks. This is achieved thanks to ZiWi’s distributed nature: the home controller hardware is kept at the bare minimum for executing real-time tasks, delegating the processing of the data and the decisions on non real-time automated tasks to remote cloud servers.

It is important to note that ZiWi proposes a distributed approach instead of a decentralized solution [29]. Both terms have been used as synonyms in some scenarios, so they can be confusing. In the case of decentralized systems, they consist of a network of stars where every central element of each star processes the collected information locally. In contrast, in a distributed system, the different components are located on networked computers that communicate and coordinate to achieve a common goal. This is what is proposed in fog computing: local gateways can communicate with each other and with the cloud to achieve a common goal [4,30]. What can be confusing is that many decentralized systems are also distributed, since they process some information locally but also cooperate with other systems to perform certain actions (e.g., this is the case of some peer-to-peer or blockchain-based systems [31]), so a distributed system can also be decentralized in some applications.

In the case of ZiWi’s architecture, the term distributed implies that the computational and storage resources are distributed throughout the network in strategic locations to allow the system to respond faster and also to decrease the computational load of the cloud. This is performed by moving part of the computational power towards the edge by means of fog gateways, which perform certain simple tasks that require a fast response.

This architecture also allows ZiWi to remain inexpensive in comparison to many commercial systems, which usually make use of complex and pricey home controllers. The designed architecture, together with the use of an open messaging protocol like Message Queuing Telemetry Transport (MQTT) to communicate nodes, make ZiWi more flexible than traditional home automation solutions, whose manufacturers are often reluctant to offer connectivity with third-party systems. Thus, such an openness and the use of widely known technologies provide robustness and ease of construction and deployment, being straightforward for integrating new elements on the ZiWi IoT ecosystem and allowing the user to make use of a wide range of resources to act on devices to obtain information on them or to automate certain events.

This article includes three main contributions aimed at creating a cost-effective and duplicable fog computing-based HAS. First, in order to establish the basics, it presents a detailed review of the state-of-the-art of the main and the latest technologies and services for home automation systems. Second, it thoroughly explains the design, implementation and practical evaluation of a fog computing-based HAS that is accessible, easy to configure, low cost and scalable in terms of protocols and technologies. Third, the article describes in detail an HAS that, thanks to the use of open-source software and Commercial Off-The-Shelf (COTS) parts, is easy to build, so it is straightforward for other researchers to replicate the system and the performance tests presented in Section 5.

The rest of this paper is structured as follows. Section 2 describes and analyzes the state-of-the-art of home automation technologies and the latest commercial and academic HAS. Section 3 includes an overview of ZiWi’s architecture and its main components. Section 4 details the hardware and software used to implement the different system devices. Section 5 presents the results of different tests that evaluate the performance of ZiWi in real scenarios. Finally, Section 6 is devoted to the conclusions.

## 2. Related Work

### 2.1. Home Automation Protocols and Technologies

There is a rich variety of protocols and technologies in the field of home automation. Some of them are standard and have been designed by international institutions, while others are proprietary and have been developed by companies. In certain cases, such protocols and technologies have been designed for wired communications, while others are aimed at creating wireless systems. Each of them presents different advantages and disadvantages depending on the deployed scenario.

Some of the most popular home automation technologies are KNX-EIB, LonWorks, X10, Insteon, ModBus, BACnet, Z-Wave and EnOcean. In addition, other generic wireless technologies like Bluetooth (IEEE 802.15.1) and WiFi (IEEE 802.11) have been used for controlling devices in home automation installations, since they provide an easy way to communicate with smartphones, tablets and PCs. Regarding WiFi, it is worth mentioning that the WiFi Alliance has recently approved a new standard for wireless connections called IEEE 802.11ah or WiFi HaLow that extends WiFi into the 900-MHz band, allowing for the connection of low-power sensor networks like the ones usually embedded into battery-powered home automation devices.

**Figure 1:**
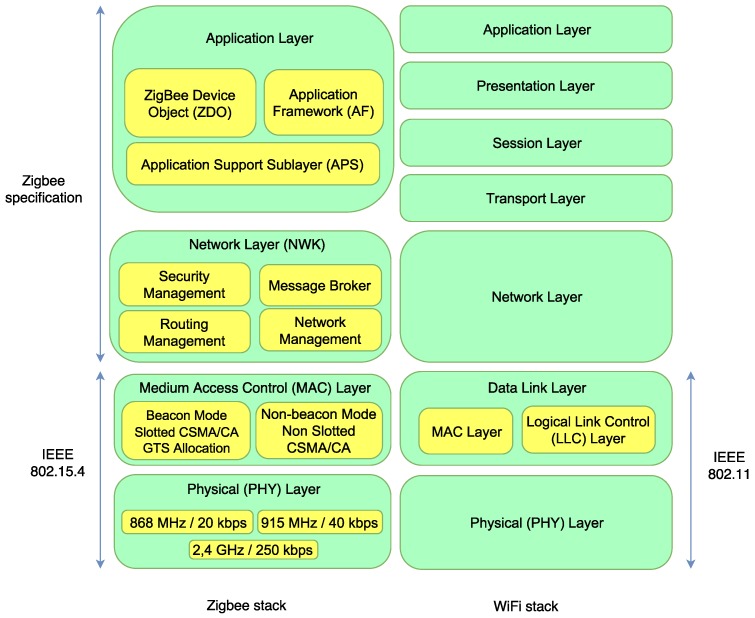
Zigbee versus WiFi protocol stack.

As it was mentioned earlier, ZigBee has to be included in the comparison, since many manufacturers have developed home automation platforms and devices that rely on it. As it can be observed in Figure 1, ZigBee’s protocol stack differs from the one used by WiFi. Actually, ZigBee nodes use Physical (PHY) and Medium Access Control (MAC) layers defined by the IEEE 802.15.4 standard, while the network and application layers are covered by the specifications given by the ZigBee Alliance [32]. In contrast, the term WiFi usually refers to LAN (Local Area Network) technologies, thus including all the layers of the stack.

IPv6 over Low-Power Wireless Personal Area Networks (6LoWPAN) is another emerging technology and, like ZigBee, it provides general purpose, easy-to-use and self-organizing wireless communications for low-cost and low-power embedded devices. Both ZigBee and 6LoWPAN implement IEEE 802.15.4 MAC and PHY layers, but 6LoWPAN has been explicitly designed for an easy interaction with IPv6-based networks, which are still far from being widely used (for instance, as of writing, less than 18% of the users access Google over IPv6 [33]). In the same way, Bluetooth Low Energy (BLE) also supports IPv6 through Internet Protocol Support Profile (IPSP), but it suffers from the same use limitations as 6LoWPAN. The latest version of Bluetooth (Bluetooth 5.0) seems to be a really good fit for low-power home automation devices [34]: with respect to the previous version (4.2), it can reach four times its range, it can transmit twice as fast, it multiplies by eight the previous broadcasting message capacity and it improves the coexistence with other cellular and wireless technologies. However, currently, there are not as many commercial home automation systems and home devices that support Bluetooth 5.0 in comparison to ZigBee and WiFi. In addition, there is a lack of commercial BLE 5.0 SDKs (e.g., Nordic nRF52840 PDK), which is a limitation when developing new hardware. Similarly, there are other good alternatives for providing low-power communications to home devices that require mesh communications (e.g., nRF [35]), but its use, even in home automation research platforms, it actually reduced [36].

Cellular communications technologies like the ones defined by 3GPP (3rd Generation Partnership Project) [37], 4G machine-to-machine communications [38] or Machine-Type Communications (MTC) [39] should also be mentioned. These technologies are still emerging and its use for home automation is actually marginal, although they have been tested in some IoT scenarios [40].

Table 1 summarizes the main features of the most relevant home automation technologies previously mentioned. For every technology, the transmission medium, the data transfer rate and whether its specification is open or proprietary are indicated. This latter parameter is essential, since a proprietary protocol, like Z-Wave until 2016, usually suffers from fewer interoperability problems than an open alternative like ZigBee, but developers are attached to the technology.

### 2.2. Academic Solutions

#### 2.2.1. Home Automation Systems for Heterogeneous Networks

In the last few years, several academic researchers have presented a number of relevant home automation systems based on the use of multi-technology devices. For example, some authors [19] studied the problem of integrating varied wireless technologies (ZigBee, Wi-Fi, GSM/GPRS) in home automation environments. Specifically, the researchers used a LabVIEW-based PC that acted as a ZigBee coordinator for collecting data from different ambient sensors (temperature, humidity, light) and that is able to control certain actuators (e.g., lighting or irrigation). Similarly, other authors made use of a PC as ZigBee coordinator in order to create an HAS based on intelligent power outlets [41].

An example of a multi-transceiver (X10, Serial, EIB, ZigBee, Bluetooth, DTMF, CAN and GSM/GPRS/UMTS) HAS is presented in [20]. In such a paper, the authors detail an indoor ambient intelligence platform and an IP-based messaging protocol to communicate the home automation controller with the rest of the equipment. Baraka et al. [21] presented a low-cost HAS based on the use of a gateway consisting of an Arduino MEGA with Ethernet and ZigBee shields, and an Android tablet that acted as home controller. The system uses ZigBee for connecting wireless sensor nodes and X10 for wired communications. An Arduino MEGA, an Ethernet shield and an Android phone are also used in [42], but, in this case, the sensors and actuators were connected directly to pins of the Arduino.

CONDE [43] is another HAS whose objective is to improve energy efficiency in smart buildings. CONDE is decentralized and thus allows for reducing response time and power consumption with respect to traditional centralized systems. CONDE was tested by using MICAz motes, which were able to lower decision delay times and increase energy savings when evaluating lighting and Heating, Ventilation and Air-Conditioning (HVAC) systems.

Other researchers have also proposed home controllers and gateways for HAS that support only WiFi [44,45,46], only ZigBee [22] or both technologies [23,24,25]. Most of them [22,23,24,25] do not use open messaging systems but proprietary ad hoc protocols, while some of the latest use MQTT [45,46] or Extensible Messaging and Presence Protocol (XMPP) [44]. For instance, in [45], it is proposed the use of low-cost WiFi modules to transform a traditional house into a smart home. For such a purpose, the authors make use of ESP8266 WiFi modules for the nodes, a Raspberry Pi 2 for the gateway, MQTT for data exchanges and OpenHAB as home automation server. Similarly, in [46], a simple HAS based on MQTT is presented, but the home controller is implemented on a PC.

Regarding ZigBee-based home automation systems, they are not very popular when having to interact directly with mobile devices, but some researchers have demonstrated that it is possible to connect Android phones/tablets to a ZigBee dongle [47].

With respect to hybrid systems, it is worth mentioning the work of Vivek et al. [25], who presented a WiFi-ZigBee gateway aimed at enabling IoT services in an HAS. The home system controller is developed on a Cubietrack board connected to ZigBee and WiFi wireless interfaces that communicate with sensor nodes that collect data on ambient parameters and that are able to actuate on relays that control lights and fans. Another example of heterogeneous HAS is MPIGate [23], which is based on a multi-protocol gateway that integrates sensors and actuators with different network protocols (e.g., EIB/KNX, WiFi, Bluetooth, ZigBee). For the sake of clarity, Table 2 and Table 3 show a comparison of the most relevant characteristics of the previously cited academic systems together with the ones of the system proposed in this article. As it can be observed in Table 2, ZiWi is the only academic solution that, at the same time, makes use of MQTT and supports both WiFi and ZigBee nodes. In addition, in contrast to ZiWi, most systems are not open source. Moreover, ZiWi is the only HAS conceived from scratch to harness the benefits of fog computing. Regarding the cost, most academic papers do not indicate the real cost of the presented solutions, although in some cases it is specified that it is low cost (without further details). Just a couple of systems calculate explicitly the cost of the system, but it is difficult to make a fair comparison, since not all the solutions provide the same amount of hardware (i.e., nodes) and features. In the case of ZiWi, the cost was calculated for the hardware used to build the demonstrator evaluated in Section 5, so it includes all the nodes and sensors described later, as well as the cost of the selected WiFi router.

It is important to note that, although there are systems where ZigBee and WiFi coexist, they suffer from cross-interference, since their radio channels overlap and, even in non-overlapping channels, out-of-band emissions can cause interference. This issue has been studied in the past [48,49,50]. For instance, in [50], it is concluded that, in order to avoid cross-interference in the 2.4 GHz band, a 20 MHz bandwidth should be left unoccupied between the operating channels.

It is also worth mentioning that there exist commercial solutions to connect WiFi and Ethernet home automation devices with the ones that use ZigBee. For instance, Digi [51] provides Zigbee to IP gateways, but they are relatively expensive (as of writing, a ZigBee-to-Ethernet gateway costs around $100).

#### 2.2.2. Fog Computing Architectures and Applications

Many recent user-centric IoT applications are latency sensitive or require real-time data analysis and decision making. Cloud computing solutions usually cannot fulfill such requirements in many context-aware applications, so, in the last few years, different researchers proposed alternative approaches and optimizations in terms of scalability, economic and environmental effects [52,53]. One such approach is fog computing, originally coined by Cisco in 2012 [4], which was introduced to alleviate some of the above-mentioned problems and to provide a solution to support geographically distributed, end-device mobile, heterogeneous, latency sensitive, and Quality of Service (QoS) aware IoT applications.

A comparison between cloud and fog computing together with a performance evaluation is presented in [54]. This article also identifies some key research directions for fog computing such as fog federation, mobile fog computing or semantic-aware fog computing. Regarding fog computing based architectures, Mukherjee et al. [55] provide a comprehensive survey of recent implementations. In the paper, it is distinguished among the following architectures:

- Generic three-tier architecture [56]: it is one of the most widely used in fog computing. It consists of an IoT node layer, a fog layer and a cloud server.
- Layered approach [57]: it consists of six layers (physical/virtualization, monitoring, pre-processing, temporary storage, security and transport layers).
- Combined fog-cloud architecture [58]: it is used when there is a different storage and computing capacity in the fog nodes. It is detailed later, since it was the fog architecture selected for implementing ZiWi.
- Virtualized fog data centers [59]: this architecture enables adding multiple virtualized edge data centers to offload services from traditionally massive data centers.
- Fog Radio Access Networks (F-RANs) [60,61]: they use remote radio units with caching and signal processing capabilities.
- Software-Defined Networking (SDN) fog architecture [62,63]: the main difference between the traditional fog architecture and an SDN-based fog is the fog-SDN controller used to support dynamic QoS.

**Figure 2:**
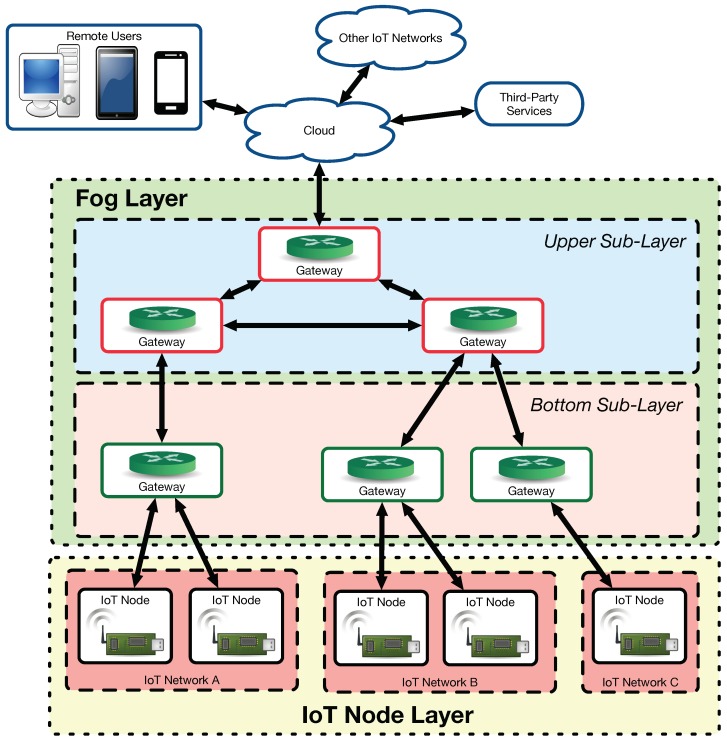
Generic fog computing architecture.

A detailed description on the inner workings of the different fog computing architectures is out of the scope of this paper, but the interested readers can find interesting overviews in [54,64]. Nonetheless, it is worth mentioning that such architectures have been used in multiple fields like smart grids [65,66], smart cities [67,68] or smart health [69]. However, with respect to the application of fog computing to home automation, just a few very recent papers describe practical implementations explicitly conceived for fog computing [70,71]. Such developments make use of an architecture similar to the generic fog computing architecture illustrated in Figure 2. In such a figure, three different IoT networks (A, B and C) exchange data with a fog layer that allows for communicating them with the services provided by the cloud. In the fog layer, there are two sub-layers. The bottom sub-layer is made of gateways that respond quickly because of their proximity to the IoT nodes. However, the gateways of the bottom sub-layer usually embed less powerful hardware than the gateways of the fog upper sub-layer. In addition, in such an upper sub-layer, the gateway at the top is the point of entry to the fog, while the other gateways provide different services or share among them certain data in order to reduce the latency response from the cloud.

#### 2.2.3. Protocol Compatibility Approaches

In order to create uniform, scalable and easily configurable systems, different researchers have addressed the compatibility issues associated with the diversity of protocols, technologies and standards that exist in the field of home automation. The solutions presented usually propose universal and open standards. Some of them use a configuration system based on the eXtensible Markup Language (XML) format that can be easily exchanged among standards [72]. Other researchers [73] suggest the use of the Universal Plug and Play (UPnP) open standard as a communications protocol for devices on a local network, since it offers technology independence and increases scalability.

The most relevant initiative for adding plug-and-play capabilities to intelligent devices based on transducers (i.e., on sensors and actuators) is the ISO/IEC/IEEE 21451 standard (previously known as IEEE 1451). The standard is highly-flexible and generic, which implies a certain degree of complexity that makes it difficult to implement in the resource-constrained devices usually found in home automation installations. For such a reason, some authors proposed modified and simpler versions aimed explicitly at home automation applications [74].

Besides standards, messaging protocols have arisen as a way for connecting heterogeneous IoT nodes. Such protocols are really useful in applications where different components are designed and implemented independently using diverse programming languages or hardware platforms, but they have to be able to communicate, collaborate in tasks, and share data among them. Traditionally, this kind of communication was performed by exchanging files, sharing a common database or by using calls to remote procedures (i.e., creating Remote Procedure Call (RPC) based systems), but, in the last few years, it finally was derived in what is called a messaging system or Message-Oriented Middleware (MOM).

A messaging system centralizes communications in order to minimize the coupling between the components of a distributed system. This decoupling and the use of asynchronous communications allow for carrying out more robust communications: the components to be communicated do not have to be working at the same time, so they delegate the delivery of the data to a messaging system, what enables them to stay focused on the information to be sent instead of on how to send it.

A messaging system is also in charge of establishing and managing the connection points and channels created among the different clients. It is usually implemented as a software process called a “messaging server” or “messaging broker”. Most messaging brokers can cooperate to provide advanced features like load balancing, fault tolerance, or sophisticated routing systems, which are really useful in IoT applications.

Messaging systems transmit data over channels that connect a transmitter and a receiver virtually. There are basically two channel models: the peer-to-peer queue model and the publish/subscribe model. The peer-to-peer queue model is currently the most used. It allows a transmitter to send a message to a queue where it will be read by a particular receiver. Note that, although the transmitter sends the message to a queue, it does not indicate who the receiver is: it only specifies the name of the queue where the data are shared. Any receiver that knows the name of the queue can connect to it and collect the message, which will be removed from the queue. Thus, the model does not guarantee that a specific receiver will always collect the data, but it does guarantee that, if someone connects to the queue and collects the message, it will only be collected once and by a single receiver.

In the publish/subscribe model, messages are transmitted to one or more recipients who have previously expressed interest in them. When the transmitter publishes a message on the channel, it sends a copy to each of the output channels. Each of these output channels has a single subscriber, which is the only one that can consume the published message once. Therefore, each subscriber receives the message only once and the various consumed copies disappear from their respective channels. There are other variants of the peer-to-peer queue and publish/subscribe models, but the ones previously described remain as the most commonly implemented in popular messaging systems (which are briefly described in the following paragraphs).

One of the most widespread messaging systems is Java Messaging Service (JMS), which is actually an Application Programming Interface (API) that is part of the Java Enterprise Edition (JEE) and it is aimed at exchanging asynchronous messages among two or more components developed with JEE. In practice, to make use of JMS, it is necessary to have a JMS provider that manages sessions and message queues. Since JEE version 1.4, the JMS provider is included in all the implementations of JEE servers, but there are other alternatives like Apache ActiveMQ, HornetQ, OpenJMS or IBM’s WebSphereMQ.

Another messaging system is Advanced Message Queuing Protocol (AMQP), which is an open standard for the development of MOMs. It is oriented towards scenarios where high performance, flexibility, security and reliable routing/delivery of messages are required. One of the most relevant features of AMQP is that it is a standard protocol and, therefore, the implemented components will be compatible. This is important, since the previous attempts to standardize the MOM before AMQP were limited to the API level (e.g., JMS) and, because of this, complete interoperability was not guaranteed. Unlike JMS, which simply defines an API, AMQP defines a complete protocol and, therefore, it includes a byte-level format description of the data sent over the network. Some of the most relevant AMQP implementations are Apache Qpid, SwiftMQ and RabbitMQ.

XMPP is another popular messaging protocol that has been used, for instance, by Google Talk or Facebook’s chat. XMPP is defined as an open standard based on XML. Originally, it was known as Jabber and it was aimed at creating a quasi-real time messaging system that allowed for transmitting data to a list of contacts. Thanks to its flexibility, it has been used for implementing file transfer systems, smart grids or social network services. Another key feature of XMPP is its decentralized architecture (there is no central server, anyone can set up his/her own XMPP server) and its security (for instance, it allows for using Transport-Layer Security (TLS) and for isolating the servers from public networks). There are numerous implementations of XMPP servers and clients, like Ejabberd, Apache Vysper, Citadel or CommuniGate Pro.

MQTT [75] is an open messaging protocol that enables the transfer of messages from ubiquitous devices (e.g., sensors, actuators, mobile phones, embedded systems or laptops) and in networks with resource constraints or high latency. MQTT’s main specification (v3.1) makes use of a publish/subscribe model that consumes very few resources, what is useful in situations where resources are restricted (i.e., when there are hardware constraints in terms of memory/capabilities or when the network speed is low). Note that there is also a v2.1 specification oriented towards networks of sensors and embedded systems operating over non-TCP/IP based networks such as ZigBee (in fact, this specification defines the MQTT-SN messaging protocol that follows a publish/subscribe model adapted to sensor networks). Some of the most relevant MQTT implementations are ActiveMQ and Mosquitto [76].

Finally, it is worth mentioning, Simple (or Streaming) Text Oriented Messaging Protocol (STOMP), which is a text-based protocol characterized by being extremely simple. Its specification is very similar to HTTP, but it is even simpler. Because of its simplicity, there are multiple STOMP implementations, like the ones provided by Apache Apollo, RabbitMQ, Apache ActiveMQ, CoilMQ or Gozirra.

The previously mentioned messaging protocols are probably the most popular, but there are also other relevant MOMs that have achieved a certain degree of popularity in specific scenarios, like OpenWire, Amazon SQS, the traditional IRC (Internet Relay Chat), PSYC, Beanstalk, ZeroMQ, Peafowl, Gearman, Sparrow, Kestrel or Apache Kafka.

Among all the different messaging systems, MQTT is arguably the most used by IoT and WSN applications because of being so lightweight that it can be implemented in resource-constrained devices like the ones usually found in HAS. Specifically, MQTT can be found in applications based on low-power sensors for smartwatches [77], in robotics [78], healthcare [79] or optical camera communications [80]. In the field of home automation, MQTT has been used in different applications to control lights [81], to monitor ambient assisted living parameters [82], for home energy monitoring [83], or for the general management of a smart home [46,84].

In academic research, the major rival of MQTT is XMPP, but most XMPP-based home automation developments [44,85,86,87,88] were published before MQTT became an ISO standard [89]. Other messaging protocols have not had relevant success in home automation, although, for instance, some researchers optimized JMS performance [90] or proposed using AMQP through RabbitMQ [91].

### 2.3. Commercial Home Automation Solutions

Currently, there is a great variety of commercial home automation systems. Some of the most popular solutions are provided by manufacturers like Qivicon [18], HomeSeer [92], Loxone [93] or Domintell [94], whose characteristics are a good reference when evaluating the technological maturity and functionality of most modern home automation systems. Such characteristics are compared in Table 4, where it can be observed that all solutions offer different communications protocols for wired and wireless transmissions. Regarding features, they support basically the same, but Loxone and Domintell do not offer a video surveillance system (for the sake of fairness, it should be mentioned that Loxone and Domintell can make use of a video intercom and that surveillance cameras could be integrated in the system and then controlled through a Virtual Private Network (VPN)). Moreover, the user experience in HomeSeer and Qivicon is considered acceptable since these systems allow for the creation of complex configurations, but such operations are really difficult to carry out by a user without certain knowledge or experience.

As far as diversity of peripherals, Qivicon is probably the most advanced solution, since their developers collaborate with numerous companies to be able to integrate a wide variety of wireless devices. HomeSeer also includes a wide variety of peripherals, but the protocols they implement are more restrictive than the ones used by Qivicon. In the same way, Loxone makes use of a proprietary protocol for wireless communications (Loxone Air), which also reduces the range of compatible devices.

It is also worth noting that, in the case of Domintell, their home automation devices are connected to analog inputs and they only have infrared transceivers, so they cannot communicate with wireless devices based on other technologies.

Finally, regarding the prices included in Table 4, note that they represent an approximate cost for a basic system. The final cost will depend on the number of devices to be installed, as well as on the size of the house and on the user needs. Thus, the minimum cost will be usually more than twice the amount indicated in Table 4.

### 2.4. Open-Source Home Automation Software

One of the objectives of ZiWi is to provide a low-cost and flexible HAS, thus open-source home automation software is perfect for the task: in most cases, it offers a free version that can be easily modified to adapt to the needs of a project.

Table 5 shows a comparison of the features of some of the most popular open-source home automation platforms. It can be observed that, although most of them are promoted as home automation systems, a minority has evolved towards IoT frameworks, which, in part, can be applied to home automation applications. The compared systems are also very similar respect to their interface (web-based), the amount of implemented protocols and their support for low-cost computer boards like the Raspberry Pi. In addition, almost all include MQTT support, HTTP-based or RESTful APIs, an extensive list of plugins and a good amount of documentation for both beginners and developers.

### 2.5. Analysis of the State-of-the-Art

After reviewing the different aspects of the state-of-the-art, it is possible to highlight several important shortcomings that motivated the creation of ZiWi. First of all, the lack of a common standard in home automation and the wide range of existing technologies and protocols imply two main problems: the difficulty of integrating all technologies in the same system and the existence of restrictions when including proprietary technologies. In fact, the use of proprietary protocols can be a relevant limitation, since they usually force the consumer to acquire only devices of certain brands.

Second, the cost associated with the deployment and maintenance of a commercial HAS is really high, and the larger the number of technologies and devices that make up the system, the more expensive and the more prone to failure it becomes. There are many factors that influence the price (i.e., house size, product versions, support) but, in any case, a basic package for a deployment in a small house does not cost less than €1000.

Third, when deploying an HAS, it is essential to distinguish between new and already-built houses. In the former, both wired and wireless devices usually can be used. In the latter, it is desirable to use wireless technologies, since wired technologies generally involve cumbersome tasks for adding infrastructure that, in some cases, might even be impossible to install. In this regard, ZigBee and WiFi are the most popular wireless standards and, although a few developments have proposed the creation of a ZigBee-WiFi HAS, none has been found that includes all the features of ZiWi and, at the same time, proposes a fog-computing architecture whose performance and cross-interference are evaluated in real-world scenarios.

Finally, the fourth shortcoming is related to the fact that, although different alternatives have been proposed for adding new home automation devices while preserving compatibility among them, most of them require complex and flexible hardware, which cannot be usually embedded into home automation sensor nodes. Messaging systems provide a good alternative for simplifying the communications between the home controller and the sensor/actuator nodes, but only a few are simple enough to be implemented in the resource-constrained devices found in many home automation systems. Among the different messaging systems, MQTT is arguably the best choice, since it has been explicitly designed for sensor nodes with limited computing power and memory. Moreover, MQTT allows for adding devices that do not implement a TCP/IP stack.

Therefore, ZiWi has been devised taking the previous shortcomings into consideration, while, at the same time, it offers the basic functionality required by a regular HAS.

## 3. System Design

### 3.1. HAS Architecture

**Figure 3:**
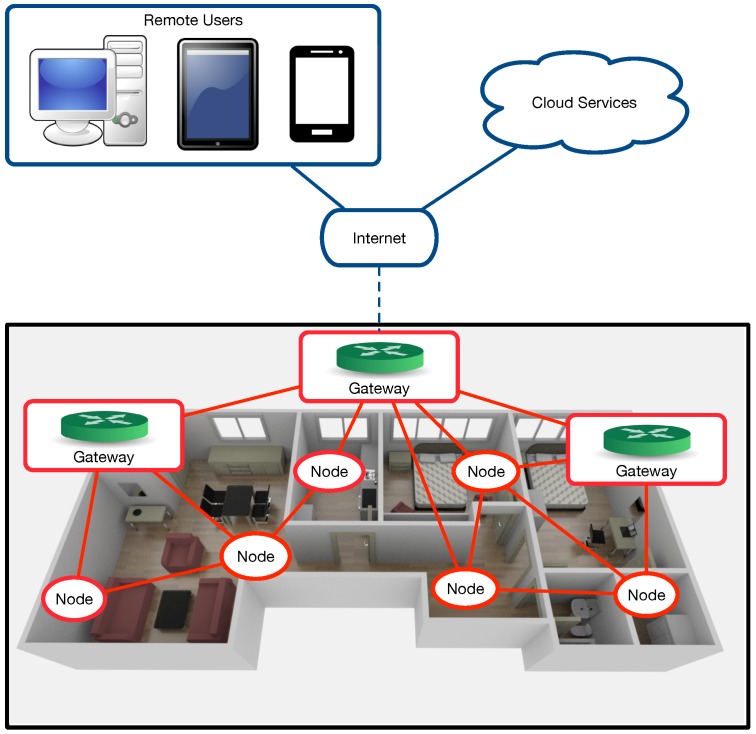
General view of ZiWi’s communications architecture.

Figure 3 depicts the proposed architecture for ZiWi, which is a single-home version of the generic home automation architecture shown in Figure 2. As it can be observed, there is a group of gateways that are installed locally on embedded devices, although the system has been designed so that the functionality provided could also be offered through external cloud services. Specifically, such a group of gateways is responsible for the HAS user interface and the communications with the different home automation devices, also providing persistent storage for the data collected from the sensors.

ZiWi’s node communications are exclusively wireless through WiFi and ZigBee transceivers. Nodes are connected either directly to a gateway or to other nodes that forward their data. Every node consists of several sensors and/or different actuators, whose number and type depends on the room where the node is deployed, existing certain common sensors/actuators to all rooms, like the ones related to temperature, humidity, luminosity or the light dimmers.

It is important to note the different nature of both types of home automation devices present in the architecture: in most home automation systems, sensors tend to be more numerous than actuators and send frequent data updates, while actuators only operate on specific occasions. For this reason, ZigBee is usually preferred for sensors (especially for the ones that depend on batteries), while actuators, which usually have to be kept listening continuously for remote commands, are equipped with WiFi transceivers that allow for receiving direct IP packets from the home controller.

The WiFi modules and the home controller communicate through the home network using a WiFi router, adding APs (Access Points) as repeaters when necessary. If the WiFi infrastructure is limited and coverage needs to be increased, the architecture would enable using ZigBee routers, which would only require connecting an XBee module to one of the General-Purpose Input/Output (GPIO) ports of the node’s embedded microcontroller.

### 3.2. IoT Nodes

There are two basic types of IoT nodes available in the system, the ones equipped with WiFi, and the ones with ZigBee. Regarding the ones with ZigBee, the choice of hardware is limited, mainly because of it is dependency on the ZigBee Alliance, which only allows the free use of the technology in non-commercial projects. Although there are different manufacturers of IEEE 802.15.4-compliant modules (e.g., Atmel’s ATZB-24-A2 or Embit’s EMB-Z2530PA) and some open-source ZigBee stacks (e.g., ZBoss [110]), the most straightforward way of making use of ZigBee is by using Digi’s Xbee modules [51], which enable using the ZigBee stack right out of the box.

The are different Xbee versions, which differ in certain characteristics (detailed below). Note that some versions are not compatible with each other, so a developer is restricted to use hardware from a specific XBee series. The different versions of XBee and their main features are as follows [51]:

- XBee Series 1. They are mainly used for point-to-point communications. They are the simplest to use and, in fact, they can be used without almost any prior configuration.
- XBee Series 2. This series has evolved through the last few years as its features have been improved, both at a hardware and at a firmware level. They allow for configuring the modules to create a mesh network, where three different roles are assigned to nodes, existing coordinators, routers and end-devices. End-devices collect data from sensors or receive remote information from other nodes. Routers mainly act as information relays for other nodes, but they can also collect sensor data or manage actuators. The coordinator acts as a gateway of the mesh network and gathers all the information from the sensor nodes.

Apart from belonging to one of these two types, each device may have the following characteristics:

- Standard or PRO version. There are few differences between a regular XBee and an XBee PRO. The main difference in hardware is that the XBee PRO is a little more complex and enables programming the module. With respect to communications, the PRO version has a longer range (according to the manufacturer, up to 1.6 km in Line of Sight (LoS) scenarios) at the expense of higher power consumption. Despite these differences, the two models can be mixed within the same network.
- Operation frequency: 868/915 MHz or 2.4 GHz. Most XBee modules operate at 2.4 GHz, but there are a few that operate in the 900 MHz Industrial, Scientific and Medical (ISM) band. The advantage of working in this latter band is that signals can go further, especially with a high gain antenna, and have greater penetration, being able to reach in some scenarios up to 24 km. However, note that, unlike most of the 2.4 GHz band, the 900 MHz band is not universal, so transmissions in such a band are not allowed in some countries. Obviously, these two models cannot be mixed on the same network.

Regarding the WiFi transceiver, there is a wide range of embedded devices in the market that allow for communicating with the various HAS devices. Until recently, WiFi-based communications were relatively expensive, but the release of transceivers like ESP8266 System on Chip (SoC) [111] (Espressif Systems, Shanghai, China) have supposed a revolution in the IoT ecosystem. The ESP8266 is really cheap (as of writing, it costs less than $2 per unit) and provides both a WiFi transceiver as well as a microcontroller that can be programmed using the Arduino coding environment. While it is true that new alternatives to these WiFi modules are rising recently, such as Realtek RTL8710 or ESP32 (Espressif Systems, Shanghai, China), they do not have the same community support and are not as widespread as the ESP8266.

It must be also noted that there are already ESP8266-based IoT home automation devices on the market like the Sonoff (Itead Intelligent Systems Co. LTD, Shenzhen, China) family of products [112], which are inexpensive and really easy to use. However, they present several limitations that also exist in other commercial IoT proprietary devices:

- The manufacturer approach for the Sonoff control application (eWeLink) relies on a cloud AWS (Amazon Web Services) server, so every request goes through the cloud. Therefore, Sonoff devices cannot perform complex actions or interact with each other without an Internet connection. In addition, all the private information on the use of home devices might be collected by a third-party.
- In the last few years, several security problems were found in Sonoff devices:
  – The original Over-The-Air (OTA) updating mechanism can be used to update remotely the official firmware [113].
  – Although Sonoff devices make use of HTTPS, they do not verify SSL certificates [114], so it is straightforward to perform man-in-the-middle attacks to sniff and to alter the exchanged information.
- The firmware of the Sonoff devices is not open-source, so the user has to stick with the manufacturer features and the previously mentioned security problems. Nonetheless, some users were able to reverse-engineer certain Sonoff devices and have developed their own ESP8266 firmware [115]. Thus, such alternative firmware would allow for using Sonoff ESP8266-based devices as regular ZiWi nodes.
- Although the manufacturer provides schematics, the potential sensors to be used are limited to the ones embedded, so the devices lack flexibility when having to add new sensors or actuators.
- As of writing, there are not Sonoff products that implement the ZigBee protocol (only WiFi, GSM/GPRS and 433 MHz RF products), so their hardware would have to be adapted to be used with external ZigBee modules.

Therefore, although there are commercial products that can be used in conjunction with ZiWi’s gateway, it can be stated, from the research point of view, that it is more flexible in terms of hardware and software to design IoT nodes from scratch by using ESP8266-based boards or by connecting external ESP8266 modules to other boards. The latter has certain drawbacks. First, the ESP8266 module itself does not integrate components such as voltage regulators or USB ports, so the communications with the development environment have to be carried out through a serial-to-USB adapter. Second, the USB adapter does not provide enough current to power the module and possible peripherals, so an external power supply is usually required. Third, the input voltage is limited to 3.3 V. Fourth, many ESP8266-based boards do not include a standard pinout so, for instance, the number of GPIO inputs varies and in some development boards is very small.

Nonetheless, most of these issues are solved by different boards that integrate ESP8266 modules, such as NodeMCU v1.0 [116], Sparkfun Thing [117], Adafruit Huzzah [118] or WeMos D1 Mini [119]. A comparison of their characteristics is shown in Table 6. Among these alternatives, the NodeMCU board was selected due to its low price (around $4 as of writing), its ability to control 5 V sensors and actuators, and the fact that it can be programmed in Lua or C through the Arduino IDE.

As it was previously indicated in Section 2.5, the communications protocol between the nodes and the home controller is implemented through MQTT, which is also supported by numerous home automation platforms. Although ZigBee does not provide a direct connection to TCP/IP networks and cannot be used directly with MQTT, there are mechanisms for its integration into the system. As explained in Section 2.2.3, there is also an MQTT variant called MQTT-SN that allows for implementing MQTT in non-TCP/IP devices, but this requires the ability to implement the protocol in the ZigBee microcontroller, which is not possible in non-PRO Xbee Series 2 modules (therefore, it would require the use of an external microcontroller). To avoid the mentioned problems, ZiWi makes use of a Master node that connects ZigBee devices to the WiFi network. The hardware of the Master node is simple, but its software is not, since it has to act like a network bridge, mapping the multiple communications between the ZigBee and the WiFi network.

## 4. Implementation

### 4.1. IoT Nodes

**Figure 4:**
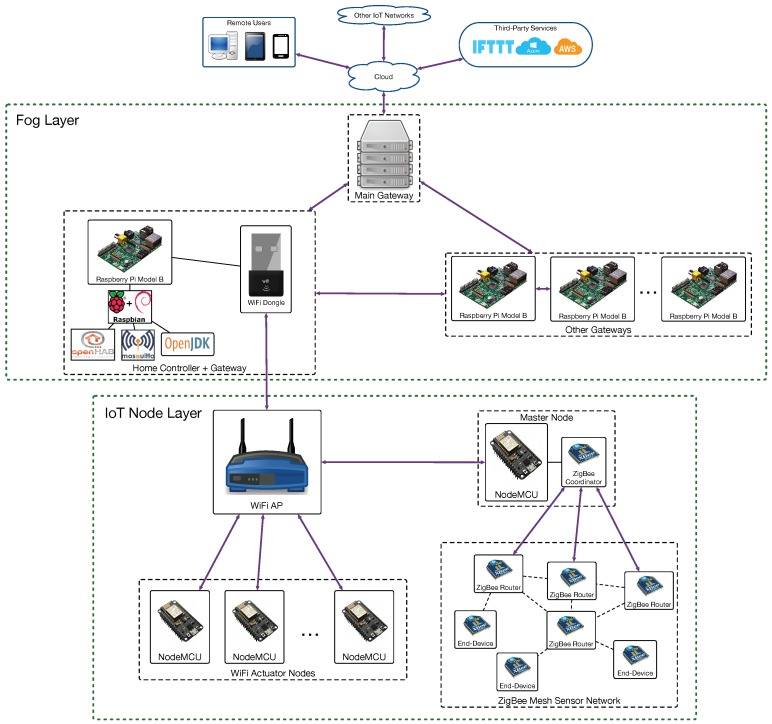
Implemented communications architecture.

ZiWi’s architecture, which is depicted in Figure 4, distinguishes among three types of nodes: sensor, actuator and Master nodes. Sensor nodes only use ZigBee to communicate, while actuator nodes make use of WiFi. Master nodes can communicate both with ZigBee nodes and WiFi TCP/IP-based devices, acting as ZigBee coordinators. In the following subsections, the most relevant aspects of the development of such three nodes are described, while all of the code of the nodes is available in GitHub [120].

#### 4.1.1. Actuator Nodes

Although in the proposed HAS architecture it is possible to use any kind of actuator, in the implementation presented in this article, only LEDs and relays are included. Relays are important in an HAS, since they are able to switch off and on electrical appliances on demand (or automatically) through messages received from the corresponding MQTT topic. Thus, during the implementation, it was necessary to determine the number of relays required by each actuator node, taking into account that each code could manage up to 11 relays (i.e., the maximum number of GPIOs available in a NodeMCU), although an external multiplexer could be added to include more.

In addition to relays, two sensors were embedded into every actuator node in order to monitor appliances. On the one hand, a current sensor (ACS712) that tolerates up to 5 A was used, which is enough to monitor appliances that require up to 1200 W. On the other hand, a temperature sensor was added to detect overheating and then prevent the current from flowing. The selected temperature sensor was an LM35, whose main characteristics are shown in Table 7 together with the ones related to the ACS712.

Note that both the ACS712 and the LM35 are analog and a NodeMCU only has a single Analog-to-Digital Converter (ADC) pin, so it is necessary to select each signal independently through a multiplexer to obtain its value. In addition, note that, when using an ACS712, the ADC actually returns an integer value between 0 and 1023 that represents a voltage that has to be processed to obtain the consumed power. For such a conversion, the value collected form the ADC is first transformed into a decimal voltage by multiplying it by 3.22 (this specific value was determined by calibrating the sensor manually with the help of a high-precision voltmeter). The value obtained represents the peak voltage. To calculate the effective current, the following formula has to be applied:

$$\begin{array}{c} I_{ef}=0.707*\frac{V_{ADC}-\alpha }{sensitivity}, \end{array}$$

where $V_{ADC}$ is the voltage read by the ADC, α represents the voltage read when there is no current flowing, and *sensitivity* is the sensitivity of the sensor according to the manufacturer. After multiple measurements, it was determined that α was 2.49 V, while sensitivity was 185 mV/A according to the manufacturer datasheet.

Once the effective current is calculated, it is only necessary to multiply its value by the contracted power (for instance, in Europe, 230 V) to obtain the consumed power.

**Figure 5:**
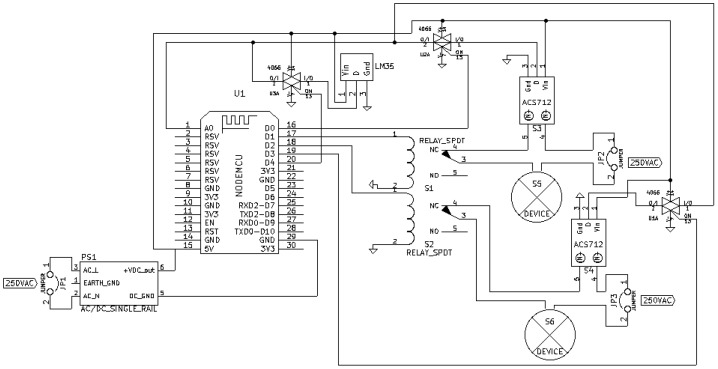
Electronic schematic of the actuator node.

Figure 5 shows the schematic of an actuator node, which contains:

- An external AC-DC converter.
- A NodeMCU.
- Two relays.
- An analog multiplexer (HCF4066BE) for choosing between the sensors that measure current (ACS172) and temperature (LM35). Note that, for the sake of clarity, instead of representing the whole integrated circuit, three of its four internal multiplexers are depicted in the schematic.
- External devices connected to the relays and to the current sensors.

**Figure 6:**
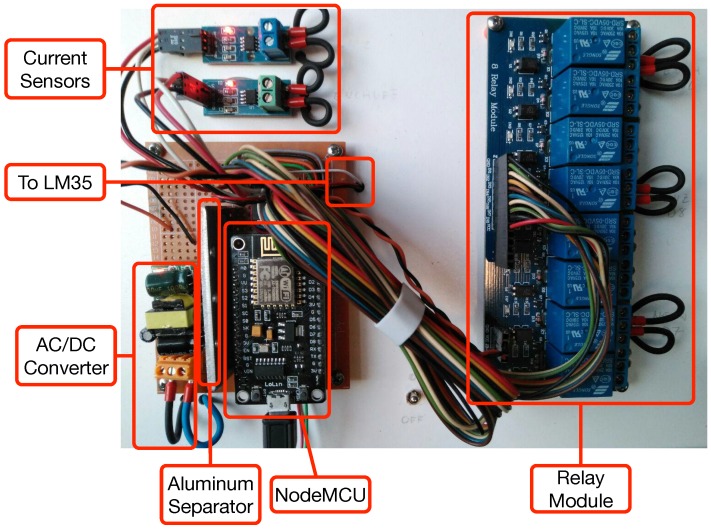
Final prototype of the actuator node.

Figure 6 shows the final prototype of an actuator node. The node is powered with a 5 V 700 mA AC-DC converter that provides enough current to power all the components and the relay module. Moreover, an aluminum separator was introduced as noise insulation screen to avoid the possible noise generated by the switched power supply, which is connected to ground to derive any possible interference. This modification was made because it was observed that the selected low-cost AC-DC converter introduced electrical noise and, since the WiFi module is close to the converter, the separator prevented potential interference in the communications.

**Figure 7:**
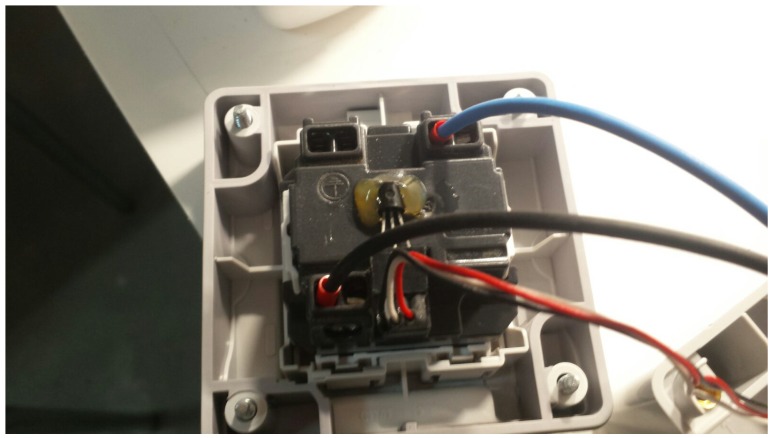
Temperature sensor glued on the back of a power outlet.

Regarding the relays, they are on a separate board and consist of eight relays that are powered through the external switched supply, since the NodeMCU is not able to provide enough current to power them. It is also worth mentioning that current sensors use three-wire connectors that are soldered directly to the corresponding pins of the CMOS analog selector, as well as to the temperature sensor. Such a sensor cannot be seen in Figure 6, since it is actually glued to the back of a power outlet, as it can be seen in Figure 7.

#### 4.1.2. Sensor Nodes

Among the different parameters to be monitored in an HAS, the most commonly measured are temperature, humidity, luminosity, electric current consumption and movement.

One of the most relevant aspects when selecting sensors and actuators for ZiWi was their operating voltage range, since they had to be adapted to those supported by the XBee and the NodeMCU. In the case of the XBee modules, they operate at a voltage between 2.8 V and 3.4 V, so the sensors’ outputs that are connected to an Xbee must operate within this range. NodeMCU modules are less problematic in this aspect, since their maximum operating voltage is 6 V, which gives more flexibility when adding sensors. Besides voltage range, another restriction when connecting sensors to an Xbee module is that they have to offer an analog output or a digital pulse to guarantee its correct operation.

Therefore, taking into account the two previous restrictions, Table 7 shows the sensors selected for implementing ZiWi, which in the case of a sensor node are:

- Relative humidity sensor: HIH-5030. This is an analog sensor that operates at a low voltage.
- Temperature sensor: TMP36. It provides an analog output, low consumption and enough accuracy for most home automation systems.
- Luminosity sensor: an LDR was selected due to the impossibility of using digital sensors like BH1750 or TSL2561. Note that an LDR is not as accurate as such digital sensors and that its output depends on the temperature at which it works. Nonetheless, the accuracy of the LDR is enough to distinguish between dark and bright scenarios.
- Motion sensor: a Parallax (Rocklin, CA, United States) Passive Infrared (PIR) sensor was chosen. This sensor outputs a high pulse when it detects movement and a low pulse in the opposite case. It is powered at 5 V and the logical output, which is the one that is connected to the XBee, reaches 3.3 V.

Besides the hardware previously mentioned, a sensor node needs additional hardware to operate. The most relevant is the power subsystem. In the case of the sensor nodes, it was decided to power the nodes with two AA 1.5 V batteries, so the input voltage is within an acceptable range for operating the XBee. Actually, the whole node is powered by three AA batteries: two of them power the Xbee and the analog sensor, while the third one is added to help to power the PIR sensor to reach 4.5 V. Note that, although the manufacturer recommends using 5 V for the PIR sensor, 4.5 V are also valid for its correct operation (however, 3 V would not be enough to let the current flow through the 3.3 V voltage regulator that the sensor requires its digital output). In this case, it would be possible to bypass the voltage regulator output to operate at 3 V (which is the minimum operating voltage), but this increases the complexity of the assembly. The only drawback of using three AA batteries is the fact that two of the batteries discharge faster since more load draws current from them. Nonetheless, the current required by the XBee, which spends most of the time sleeping, and the other sensors is really small, so, in practice, there are actually not relevant differences between the batteries discharge curves.

The hardware needed by the motion sensor is also important: it requires an inverter in order to wake up the XBee module to which it is connected, since, when motion is detected, a low signal must be sent to the Xbee module to wake it up.

Finally, it is worth pointing out that the XBee Series 2 has its reference voltage set at 1.2 V for the ADC inputs, which means that values higher than 1.2 V would always be read as 1024 (0xFFFF) and, therefore, measurement information can be lost. This is a relevant problem for the analog sensors of the node, which are powered at 3 V, since more than twice the voltage range is lost. However, note that, in most cases, the full voltage range is not used. For instance, in the case of the TMP36, when powering it at 3 V, 1.2 V are encoded approximately as 70 °C, which is a temperature that will not be reached in practice by most HAS. Nevertheless, other sensors like the ones for measuring the relative humidity and luminosity, cannot neglect the voltage range limitation, since they require a much wider range. For such cases, a voltage divider could be included at the output of the sensors, which moves the 0 V to 3 V range to an output between 0 V and 1.2 V. If sensors actually need more precision, external hardware would have to be added to the node and, therefore, current consumption and economic cost would be increased. The most straightforward solution would consist of adding an external microcontroller and/or an ADC (if the microcontroller’s internal ADC has not enough precision), and then feeding the collected data into the Xbee module through the serial interface. In addition, it should be pointed out that battery load may influence both the measurements and the calibration of the sensors. The impact of the battery load also depends on other factors like the specific sensor model or the existing environmental conditions, so further proper, well-designed experiments would be needed in order to evaluate the accuracy of the sensors through time.

**Figure 8:**
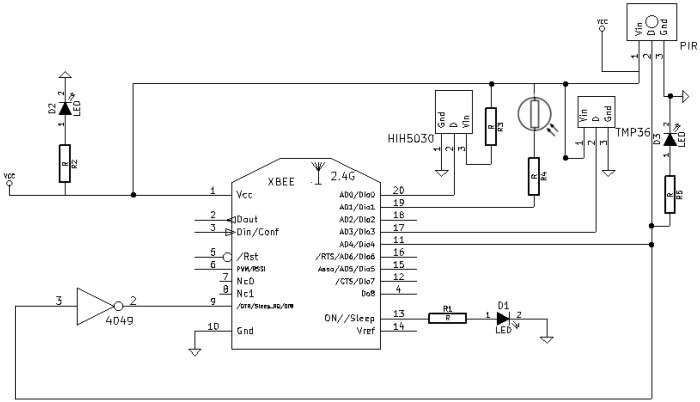
Schematic of the sensor node.

**Figure 9:**
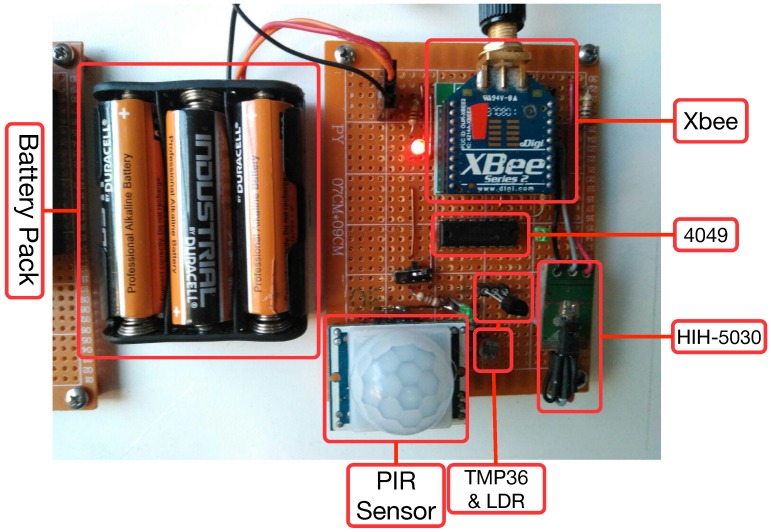
Prototype of the sensor node.

Figure 8 shows the schematic of a sensor node, which basically includes the previously mentioned components and a couple of LEDs (one for determining the status of the Xbee module and another one for debugging the operation of the PIR sensor). An actual prototype of a sensor node is shown in Figure 9.

#### 4.1.3. Master Node

The communications subsystem of a Master node is composed of a ZigBee and a WiFi module. Regarding the ZigBee module, the PRO version was not used in the HAS, since it is more expensive, consumes more power and, in ZiWi’s design, the necessity for developing more features than the ones included in the default Xbee firmware was not planned (it is enough to configure properly the network and the analog inputs). As for the operating frequency, 2.4 GHz was chosen, although it would be straightforward to replace the Xbee modules with others working in the 900 MHz frequency band if the environment presents propagation problems. In relation to the WiFi modules, a NodeMCU module was used.

**Figure 10:**
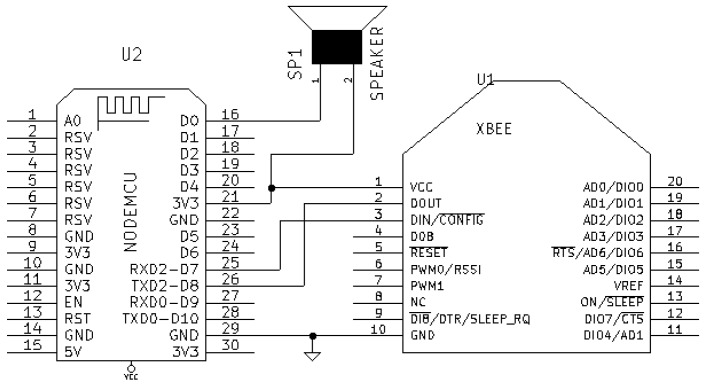
Electronic schematic of the Master node.

**Figure 11:**
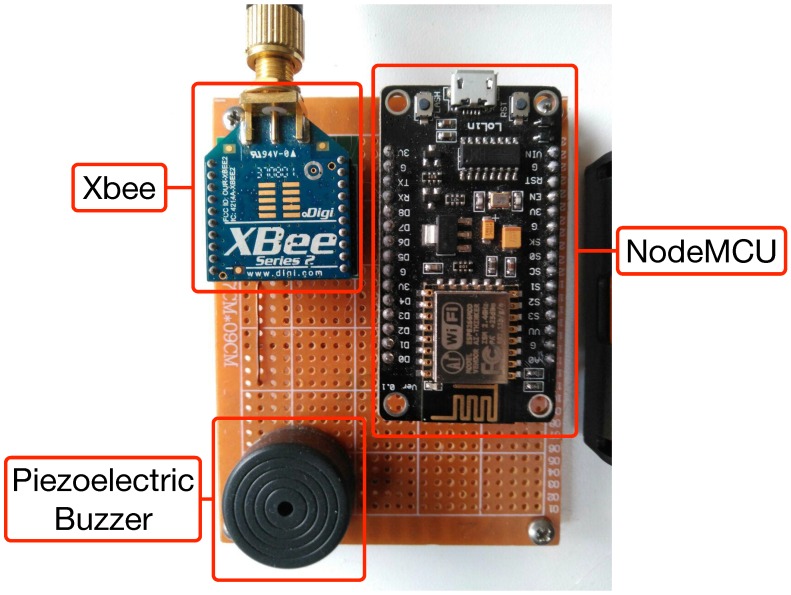
Prototype of the Master node.

Figure 10 shows the Master node’s electronic schematic, which details how all hardware components are connected: the NodeMCU and the XBee module exchange data through a serial connection, a piezoelectric buzzer is used as an alarm, and a power connector provides current through the micro-USB port of the NodeMCU. Figure 11 shows an actual prototype of the node.

Regarding the software developed for the NodeMCU, it is aimed at performing two tasks as Master node:

- To establish a serial communication with the Xbee module that acts as ZigBee coordinator. Note that in Figure 10 the pins RXD2 and TXD2 of the NodeMCU are used because RXD0 and TXD0 are dedicated to the serial connection of the NodeMCU with the USB. The frames received from the ZigBee network consist of several bytes that include information regarding different ZigBee fields. For example, a ZigBee frame generated by a node that collects data from a sensor connected to a single ADC input would be: 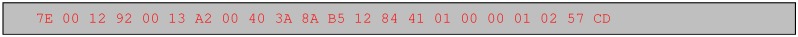 where 7E is the start delimiter, {00,12} indicate the length of the payload (that is 18 in decimal and it does not include the checksum), 92 is the frame type (in this case, IO Data Sample RX Indicator), {00,13,A2,00,40,3A,8A,B5} is the 64-bit source address, {12,84} is the 16-bit source address, 41 indicates the receive options, 01 is the number of samples collected from the ADC, {00,00} is the digital channel mask, 01 is the analog channel mask, {02,57} is the actual value read from the ADC, and CD is the checksum.
- Once the ZigBee frame is processed and the sensor data are extracted, it is necessary to convert them into the appropriate magnitude, a process that is specific for every kind of sensor.

In relation to the communication mechanism with MQTT, it consists of publishing periodically the values of the sensors using a timer.

### 4.2. Home Controller (Main Local Gateway)

The home controller is responsible for establishing communications with all the modules and the user. Its hardware requirements are actually reduced, since it only has to be able to host and run the necessary software for the server and offer a communications interface with the home network. Considering these restrictions, almost any modern computer connected to the network could be used as home controller. Nevertheless, there is a wide range of embedded devices called Single Board Computers (SBCs) that usually include a processor with the necessary processing power, storage capacity, small size, low-cost and reduced power consumption. Thus, there are devices like the Raspberry Pi Zero, the Orange Pi One or the CHIP that can be connected to the home network both through an Ethernet port or a WiFi adapter. There are also more powerful and less restricted devices like Raspberry Pi, Beagle Bone, Banana Pi or Intel Galileo. Among all these alternatives, the Raspberry Pi 1 model B+ was selected. While it is true that there are more recent hardware versions, this model has enough resources to respond quickly to events and requests that occur in a regular HAS. As an inconvenience, it is worth noting that it presents larger boot times than other SBCs when loading certain services, although, in practice, such services are not restarted frequently.

It is important to note that ZiWi was designed to minimize the computational load on the IoT nodes, so the main software components are executed on the home controller. Among the open-source home controller software analyzed in Section 2.4, OpenHAB [107] was selected due to the following reasons:

- It can run on any device capable of using a Java Virtual Machine (JVM).
- It supports a really large number of technologies, so the number of potential devices to be integrated and combined to carry out home tasks is huge.
- A growing community provides support to many ARM-based SBCs (e.g., Banana Pi, ODROID, Cubieboard...), while other platforms are focused almost exclusively on the different versions of the popular Raspberry Pi.

However, note that OpenHAB was not conceived for providing fog computing applications, but its main features make it ideal for providing home automation fog services through local gateways:

- It is designed to be completely vendor- and platform-neutral, without the need for using specific hardware or protocols.
- It has a powerful rule engine.
- It offers a flexible web-based user interface for PCs and mobile devices with different types of interfaces for managing dashboards, reports, configurations and performance benchmarks.
- It provides APIs for the integration of systems that are easily extensible, enabling the addition of new systems and devices.

Thus, OpenHAB, together with OpenJDK 8 and an MQTT broker (Mosquitto [76]) are deployed in the home controller in a Raspberry Pi that runs Raspbian, a Linux distribution based on Debian that has been adapted to run on such an SBC. For the experiments performed for this article, the last stable version of OpenHAB 2 was used.

Among the wide range of extensions that OpenHAB offers, it was decided to use Astro binding (it obtains information about the position of the sun), Exec binding (it allows for executing commands), MQTT binding (it offers support for the MQTT protocol and for the integration of the broker), Network binding (it displays information on the various devices connected to the IP network), NTP binding (it uses an Network Time Protocol (NTP) server to obtain the system date), Yahoo Weather binding (it allows for accessing weather information), MQTT persistence (it enables storing persistently the events registered by the broker), RRD4j persistence (it stores persistently the various states of the devices), Telegram action (it allows for sending notifications through a Telegram bot), Exec transformation (it is used to execute external programs), Map transformation (it obtains locations from coordinates), Regex transformation (it allows for working with regular expressions), Basic User Interface (UI) (it is a basic interface for the visualization and interaction with the devices), Paper UI (a configuration interface for the different elements of the platform), Habpanel (an interface for the creation and visualization of dashboards), Habmin (it is an advanced interface that allows for the creation of rules, graphs, groups and is also able to configure the different system elements), Google Calendar Scheduler (it allows for sending events to the nodes of the HAS through Google Calendar), my.OpenHAB (OpenHAB remote service that displays the status information of the devices of the HAS, and their events and notifications) and Rest Documentation (it provides information about the OpenHAB API).

**Figure 12:**
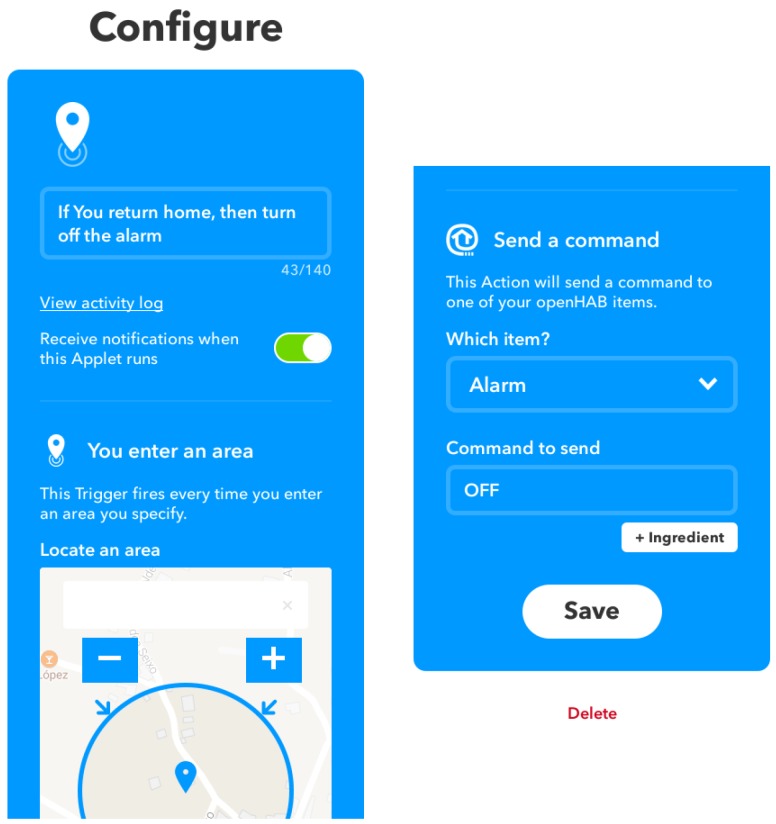
Example of the creation of a rule using IFTTT.

Regarding the remote service my.OpenHAB, it is especially interesting, since it allows for storing information about different devices persistently in a cloud, as well as their status and notifications. This remote service also enables making use of the IF-This-Then-That (IFTTT) dynamic rule creation platform [121], which is really useful for integrating the behavior of multiple objects through rules. A practical example of an IFTTT rule could consist of automatic activation of a security alarm when someone leaves his/her home and its automatic deactivation before the user’s arrival. To build this rule, it is possible to use the Android location service, which provides the location of the user and defines an approximate area on a map that represents the location of the house. When an Android phone carried by the user leaves the house, a command is sent to OpenHAB, which activates the security alarm. The deactivation of the alarm would be performed in a similar way, disabling the alarm as soon as the mobile phone enters the user’s home. The process for creating the rule is illustrated in Figure 12.

The following are just a few examples of IFTTT rules that were tested in the proposed HAS:

- Lighting was configured intelligently by making use of the luminosity sensors.
- Heating was switched on or off depending on indoor ambient temperature.
- Telegram notifications were sent when certain actuators changed their state.

**Figure 13:**
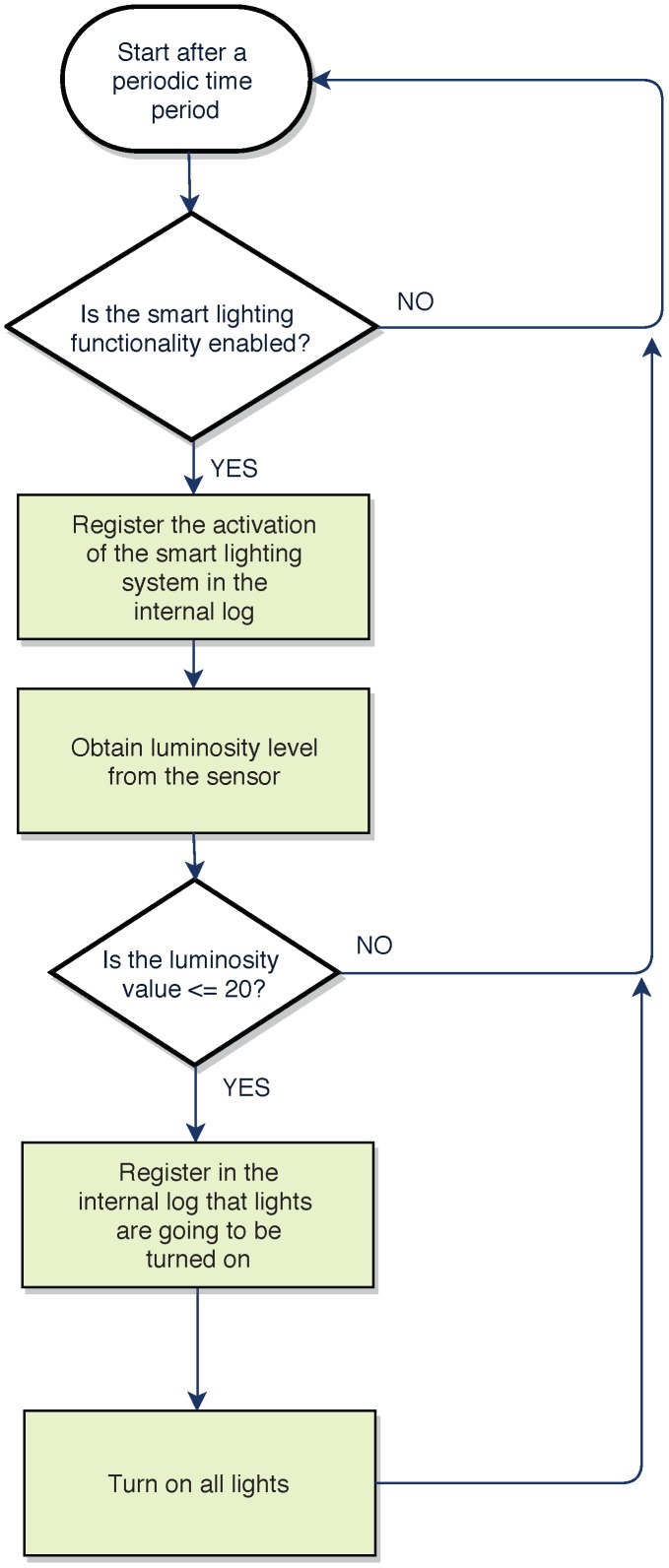
Flow diagram of the simplified IFTTT smart lighting rule.

Finally, it is worth noting that, although the fog gateway was designed to be deployed as easily and fast as possible, certain OpenHAB plugins require to create scripts in different pseudo-languages. For example, the smart lighting system detects light levels and decides whether the lights should be turned on or off with the script shown in Listing 1. Figure 13 shows a flow diagram that illustrates step by step the different tasks performed by the code. Note that, for the sake of clarity, the script and the flow diagram have been simplified, so it is only included the logic required to turn on the lights. Similar scripts are used for the definition of the events triggered by the Google Calendar Scheduler or the rules that control the Telegram’s bot.

**Listing 1.** Script for the smart lighting control rule. 
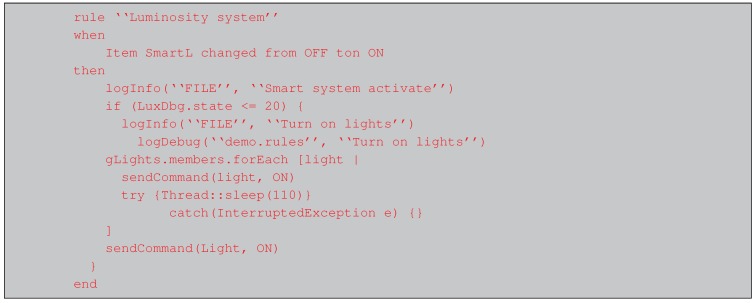


## 5. Experiments

### 5.1. Initial Configuration

There are different parameters that have to be configured before the ZiWi HAS starts operating. First, NodeMCU WiFi nodes need to be connected to the local network. To perform such a task, the network parameters of each NodeMCU need to be configured manually through a web menu in order to connect to the MQTT broker. For such purpose, the node is set to Access Point (AP) mode, so that it can be accessed with a regular web browser through the static address 192.168.4.1. The web interface allows for defining the WiFi AP (or APs) that the NodeMCU can connect to and the IP address of the MQTT broker. For simplicity, the router that acts as AP is configured to assign static IPs to each node outside the Dynamic Host Configuration Protocol (DHCP) range. In addition, a topic can be indicated through the NodeMCU web menu in order to report the status of the connection. Once such parameters are configured, the node will switch to Station (STA) mode and will start to communicate with the network.

ZigBee nodes also have to be configured. Specifically, XBee modules need to be configured in such a way that all devices in the defined mesh can communicate. This means that at least one of the nodes has to be the coordinator, while the rest will be end-devices that will send information from the sensors to the coordinator. In order to process the packets received from end-devices, the coordinator has been programmed with an API-based firmware. Such a firmware allows for using Digi’s XCTU application (6.3, Minnetonka, MN, United States) to analyze the information inside each frame easily and then verifies that the transmitted information arrives correctly.

Finally, it is worth mentioning that sensors should be calibrated before operation (and every now and then) in order to preserve the accuracy of the data:

- Temperature sensors. Ideally, they should be calibrated with an industrial temperature thermometer. It is not necessary to calibrate the whole operating range, but the temperature curve between 10 °C and 30 °C should be obtained and corrected for the installed temperature sensors, since the most common values in an HAS occur in such a range.
- Relative humidity sensors. They are calibrated like temperature sensors, by using a calibrated industrial instrument. In this case, the values between 50% and 80% should be as accurate as possible, since they are the most common in comfortable homes.
- Luminosity sensor (LDR). The accuracy of this sensor is conditioned by the operating temperature (manufacturers usually only include in the datasheet curves for a temperature of roughly 25 °C). However, since the objective of this sensor is to obtain a coarse estimation of the level of clarity or darkness, a fine calibration is not necessary. Therefore, to verify the correct operation of the sensor it suffices to check the sensor under a very low light and observe that the voltage decreases clearly. In addition, the sensors should be verified when exposed to direct light, when voltage should increase.
- Movement sensor (PIR sensor). This sensor is digital and its measurement procedure is straightforward: it outputs a high pulse when movement is detected and a low pulse when it is not. Nevertheless, it is possible to calibrate its two potentiometers: one of them adjusts the distance and the detection angle, while the other one indicates the triggering time when it detects movement (i.e., the time during which the sensor outputs a high pulse after detecting a movement, which in many scenarios should be low enough to detect two consecutive movements in a short period of time).
- Current sensor. To calibrate this sensor, it is necessary to determine the value of the voltage read when there is no current flowing. This is determined by using a high precision voltmeter. In addition, the actual consumed power was calibrated with commercial appliances (a 75 W light bulb and a 900 W hair dryer) when operating them at maximum power and then determining any possible offset.

### 5.2. Demo Prototype

**Figure 14:**
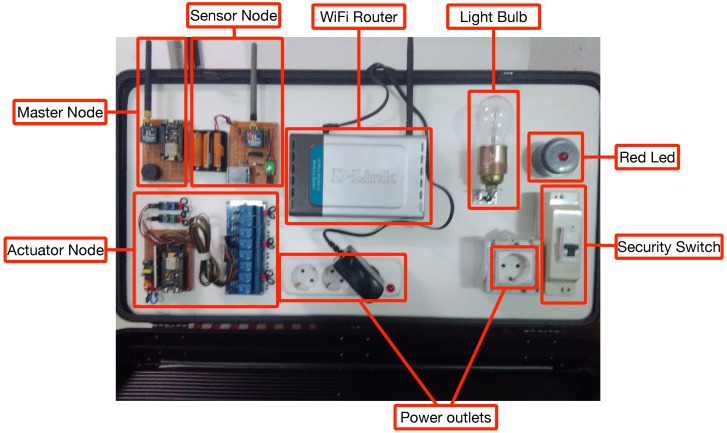
HAS prototype for showing purposes.

**Figure 15:**
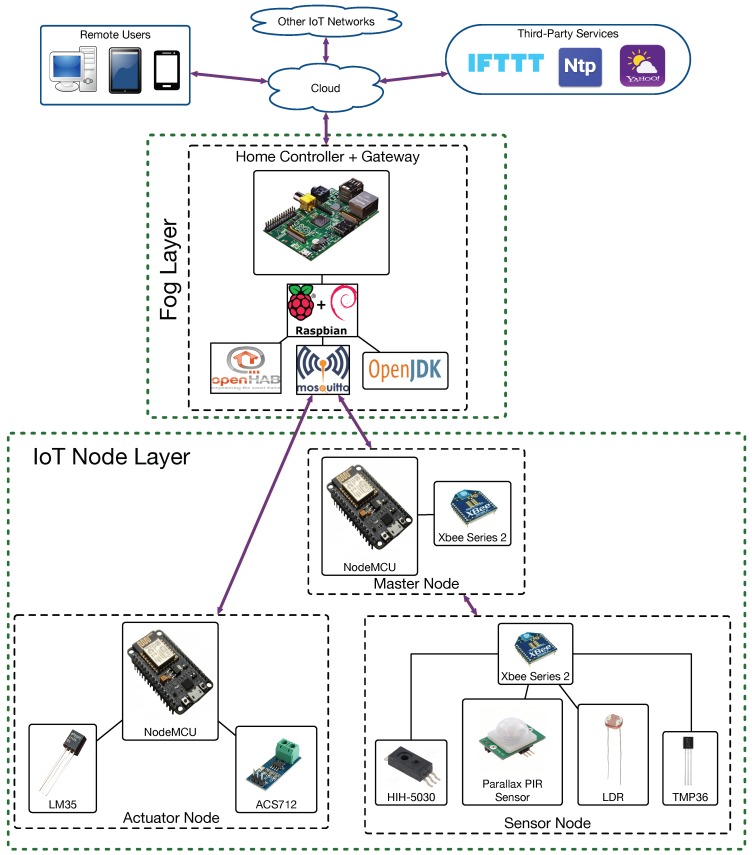
Communications architecture for the demo tests.

After calibrating the sensors, they were embedded into a demo prototype located in a real environment in order to perform different experiments. Thus, a demo HAS that included the following elements (most of them are shown in Figure 14) was built, which were connected according to the architecture shown in Figure 15:

- Sensor, actuator and Master nodes.
- A home controller (it is not shown in Figure 14).
- A WiFi router.
- A security switch to turn on and off all the elements of the system together.
- Several power outlets.
- A 75 W light bulb and a large red LED.
- Multiple LED lights to simulate the activation and deactivation of different elements.

Note that, although the implemented architecture is the one shown previously in Figure 4, during the experiments, in order to isolate the impact of the interference between ZigBee and WiFi nodes, mesh communications are not performed, and, as it can be observed in Figure 15, only a ZigBee Coordinator and an End-node communicate with each other.

### 5.3. Response Time for Actuators and Events

One of the advantages of a fog computing system is the reduction of the response latency. With ZiWi, it is straightforward to compare the difference in response time between OpenHab (the home automation service in ZiWi’s fog) and remote cloud services like Telegram and IFTTT.

Regarding the fog response, the time to turn a light on was measured when the LDR detected low luminosity, and an average of 0.02 s were measured. The average time to activate a relay was very similar: 0.018 s for switching it on and 0.016 s for switching it off.

With respect to the use of third-party services running in a remote cloud, the time required by Telegram to send messages with every relay commutation was first measured. The results showed that the average time to receive the messages in a smartphone making use of push notifications was 2.735 s. In the case of IFTTT, a rule that checked ZiWi’s luminosity every 30 min was configured and, if the values received were too low, it turned a light bulb on. In such a scenario, it was determined that an average of 51.388 s went by since the rule was checked until the light was turned on.

The obtained results allow for concluding that fog services react clearly faster than cloud services. In the case of Telegram notifications, they are roughly 160 times slower than fog event detections, while such events are 2569 times faster than IFTTT when turning a light on when detecting low-luminosity. Nonetheless, note that the experiments were performed in very specific circumstances, so, although these first measurements are promising, a detailed analysis should be performed. In addition, note that cloud services offer many complex and compute-intensive applications that would be difficult to run smoothly in a simple fog layer so, for every service conceived, the IoT designer has to look for a good trade-off between response time and functionality.

ZiWi response times can be compared to the ones obtained by other state-of-the-art systems, but it is important to note that it is difficult to compare them in a fair way due to their differences in architecture, topology, hardware, protocol compatibility or communication transceivers (as it is was previously described in Section 2.2.1). Thus, the system presented in [19] requires a 2 s delay for executing commands on an actuator in an indoor scenario (using the ZigBee network), while 25 s are required outdoors (using a GSM interface). In [25], it is indicated that 0.5 s are needed both for reading sensor values and for executing commands on actuators. In addition, other authors measured response times for actions performed by the sensor nodes (without relying on upper layers), which clearly reduces response time, but usually increases the computational requirements of the nodes. Examples of such a kind of response time are measured in [41,43], where 1233.19 µs and 32.9 ms were needed to detect events and react to them. As a summary, Table 8 compares all the previously mentioned response times.

### 5.4. Cross-Interference Evaluation

In a system where two different technologies share a common radio spectrum but that are not synchronized to avoid collisions, it is interesting to determine how well the system performs in real scenarios with and without cross-interference. However, please note that, for the sake of brevity, this article is not aimed at presenting a deep analysis of all the possible cross-interference scenarios, but at showing several relevant cases in order to determine the major factors that influence such a cross-interference in the proposed HAS. Specifically, it has been measured ZigBee’s success delivery rate for 100 packets with and without WiFi transmissions on channel 1 (centered at 2412 MHz, which overlaps with IEEE 802.15.4 channels 11 to 14).

Table 9 shows ZigBee’s packet delivery success rates for several scenarios where WiFi cross-interference existed at different degrees. Two main scenarios where distinguished: one at a relatively short distance (less than 2 m) and another one at a medium distance (approximately 10 m). In such scenarios, the WiFi network was first disabled physically to isolate the ZigBee network (there were also no other WiFi networks on channel 1), and then it was enabled. Two situations were distinguished after enabling WiFi transmissions: one where the WiFi network operated in a non-overlapping channel, and another one where it made use of the IEEE 802.11 channel 1.

The values shown in Table 9 indicate first that the collisions associated with cross-interference influence the number of received packets, the number the transmission errors, and the number of packets lost during the tests. Thus, as it could be predicted, the higher the cross-interference, the lower the packet delivery success rate. Moreover, it is interesting to point out that the mere enabling of the WiFi, although working in a non-overlapping channel, influences ZigBee communications performance due to the transmission power that is leaked to neighboring channels.

In addition, it is worth mentioning that the results presented in Table 9 were obtained with the typical traffic in a WiFi home automation network, which is light in comparison to, for instance, a WiFi network where P2P communications are carried out. In fact, during the experiments it was clearly observed that ZigBee’s packet delivery success rate plummeted as the amount of WiFi traffic rose, until reaching a point when ZigBee communications became completely jammed. Nevertheless, note that such a scenario is not usual, but the results indicate that the operating frequency has to be carefully planned in order to maximize the packet delivery success rate. For such a purpose, fog computing can be extremely helpful, since the fog layer can coordinate home gateways with the objective of synchronizing the transmission frequencies of the different neighboring devices to reduce collisions.

### 5.5. Current Consumption with Encryption

Security is often neglected, even in commercial systems [122,123], mostly because resource-constrained device are usually not powerful enough to handle secure communications protocols [124]. In the experiments performed with ZiWi’s nodes, the effect on the current consumption was analyzed when making use of encryption while communicating a node with a local gateway. Specifically, in the case of the NodeMCU-based modules, TLSv1/SSLv3 was used with ECDHE-RSA-AES128-GCM-SHA256 as cipher suite, while in ZigBee-based nodes, Application Support Sublayer (APS) encryption was enabled. The code differs slightly for the NodeMCU nodes that use SSL, so it has been also uploaded to a different folder in Ziwi’s GitHub repository [120].

Current measurements were obtained by using an Arduino and an Adafruit INA219, which allows for measuring up to 26 V and that provides enough precision (it can work in high-precision mode to measure 0.1 mA steps with a maximum of ±400 mA or, in low-precision mode, can make use of 0.8 mA steps with a maximum of up to ±3.2 A).

Table 10 shows the average current consumption obtained with and without making use of encryption in sensor and actuator nodes. In the case of sensor nodes, it can be observed that the use of Secure Sockets Layer (SSL) generates an additional computational and communications load that increases the average consumption through time, deriving into the clear differences shown in Table 10. However, the use of a lighter (and less robust) encryption mechanism like ZigBee’s APS barely increases the average consumption, but increases privacy and security with respect to not using any encryption at the nodes. Therefore, developers have no excuse for not securing ZigBee nodes using APS, but alternative energy efficient security mechanisms have to be further studied in order to improve or replace traditional cipher suites for SSL communications.

### 5.6. Key Findings

The design, implementation and practical evaluation of ZiWi allowed for obtaining diverse relevant findings that are worth being summarized for future HAS developers:

- The use of open-source software and COTS parts is essential to guarantee that the HAS can be replicated by third-parties. This is a key advantage over other academic and commercial systems, which are based on proprietary hardware or software, thus making it difficult to corroborate the obtained experimental results.
- Since ZiWi was conceived from scratch to be implemented on a fog computing architecture, it is really easy to scale it by only adding gateways. This usually occurs in two situations: when the number of home devices is too large to be handled by a single gateway, or when the wireless range provided by the fog gateways is not enough to cover the whole home or building. In such situations, fog gateways provide service redundancy and are able to communicate among them to route the data to the cloud.
- The use of OpenHAB resulted in an HAS that is really easy to manage through an attractive GUI and that is able to use a great deal of plugins to automatize many home automation tasks.
- The use of IFTTT makes it easy to connect the multiple devices deployed throughout a home, thus being able to automate the detection the relevant events and making it ideal for context-aware applications.
- The proposed system is able to decouple the hardware and software from the cloud, which reduces latencies remarkably, especially for real or quasi-real time applications (e.g., when opening doors or turning on certain appliances).
- The use of fog gateways offloads a relevant number of tasks from the cloud and also increases security. Such a security is essential for preserving the privacy of the user data, which do not have to be sent to the cloud and that do not have to be stored in servers maintained (and secured) by third-parties. In addition, device access and data availability depend mostly on fog gateways, so the communication blackouts that occur in the cloud have a limited impact on the HAS.
- The use of MQTT enables addressing most of the compatibility issues associated with the diversity of protocols, technologies and standards that exist in the field of home automation. In addition, MQTT consumes very few computational resources, so it can be implemented on resource-constrained devices.
- ZigBee-WiFi cross-interference can be problematic in environments where a lot of data are exchanged, but, in most home networks, under regular use of the communication resources, although packets can become corrupted, most of them should arrive correctly. Nonetheless, a careful frequency planning is recommended to minimize interference.
- The cost of the whole demonstrator (indicated in Table 2) is less than 20% of the cost of a basic commercial system (around €1000). Therefore, ZiWi not only is able to add numerous features, but also opens the field of home automation to many people that cannot afford costly commercial solutions.

## 6. Conclusions

This article presented ZiWi, a low-cost IoT fog computing HAS that allows for carrying out seamless communications among ZigBee and WiFi nodes. Since ZiWi makes use of open-source software and COTS hardware, it allows other researchers to replicate the fog computing architecture and then validate it. The diverse elements of such an architecture were described, showing its high scalability. Moreover, the use of protocols like MQTT allows for including resource-constrained devices in the system that act as sensors or actuators. Furthermore, the design and implementation of the Master nodes, which communicate ZigBee and WiFi devices, were detailed.

Although a more in-depth analysis should be carried out, the latency measurements obtained in several significant scenarios show that the fog computing approach can be harnessed for providing real-time or quasi-real time responses. In addition, the results indicate that cross-interference has to be taken seriously into account in environments where WiFi and ZigBee devices coexist. Regarding the performed power consumption measurements, they allow for concluding that the addition of robust security, which is essential in modern communications, derive into a remarkable increase in current consumption that should be addressed by hardware manufacturers and software developers in the next generation of IoT fog computing applications. 

## Figures and Tables

**Table 1:** Characteristics of the most common home automation technologies. RF: Radio Frequency, EG: Electric Grid, OF: Optical Fiber, TP: Twisted Pair.

Technology	Medium	Openness	Data Rate
KNX	EG, RF, TP	Open	9.6 Kbps
LonWorks	EG, RF, OF, Coaxial, TP	Open	1.25 Mbps
X10	EG	Open	60 bps
Insteon	EG, RF	Proprietary	38.4 Kbps
ModBus	TP	Open	RTU: 19.2 Kbps-TCP: 10/100/1000 Mbps
BacNet	TP	Open	10/100/1000 Mbps
Z-Wave	RF	Partially open (open-source layer for integration)	9.6 Kbps
EnOcean	RF	Partially open (open OSI layers 1–3)	25 Kbps
ZigBee	RF	Open	256 Kbps
WiFi	RF	Open	600/54 Mbps
Bluetooth	RF	Open	1 Mbps

**Table 2:** Comparison of the main features of the most relevant academic HAS and the proposed system (part 1).

System	Main Objective	Messaging Protocol	Actuation Capabilities	Open-Source Code	Conceived for Fog Computing	Cost	Relevant Features/Challenges
ZiWi	MQTT-based HAS	MQTT	Yes	OpenHAB (Node source code available on GitHub)	Yes	180 (whole demonstrator)	High flexibility, interoperability and scalability
[19]	Intelligent building monitoring	Ad hoc	Yes	No	No	Low cost	Delays due to SMS-based commands
[20]	Indoor ambient intelligence monitoring	Ad hoc	Yes	No	No	Cost-effective	Alarm control center, several scenarios
[21]	Energy efficiency	Ad hoc	Yes	No	No	Low-cost	Heuristic scheduling algorithm
[22]	ZigBee-based HAS	Ad hoc	No	No	No	Not specified	Software designed of the coordinator and terminal node
[23]	Gateway for assisted living applications	Ad hoc, dependent on the assisted living device	Yes	No	No	Not specified	Biometrics and actimetry for assisted living
[24]	HAS for heterogeneous networking	Ad hoc	Open API	No	Cloud capabilities	Not specified	Integrated home appliances with prediction algorithms
[25]	Enabling IoT services in HAS	Ad hoc	Yes	No	No	Not specified	Basic GUI with sensor readings
[41]	Power outlet control and monitoring	Ad hoc	Yes	No	No	45 (one smart socket)	Experimental analysis with theoretical and empirical measurements
[43]	Smart building energy efficiency monitoring	Ad hoc messages routed with CTP (Collection Tree Protocol)	Yes	No	Decentralized architecture	Not specified	Decision-making manager and integration of different applications
[44]	HAS	XMPP	Yes	Openfire	No	Low-cost	Android app for control units
[45]	HAS	MQTT	Yes	OpenHAB	No	Cost-effective (less than $60 for a Raspberry Pi 2, an SD card and four ESP8266 modules)	Overall delay from UI to Node is less than 600 ms
[46]	MQTT-based HAS	MQTT	Yes	No	No	Not specified	It makes use of ESP8266 WiFi modules

**Table 3:** Comparison of the main features of the most relevant academic HAS and the proposed system (part 2).

Reference	Home Controller Hardware	Communication Transceivers	Communication Topology	Sensors and Actuators	Node Hardware
ZiWi	Raspberry Pi Model B	WiFi, ZigBee	Mesh	Temperature (LM35, TMP36, DHT11), humidity (HIH-5030, DHT11), luminosity (LDR), motion (Parallax PIR rev. A) and current sensors (ACS712)	NodeMCU (ESP8266), Xbee Series 2
[19]	PC	ZigBee, WiFi and GSM/GPR	Star	Temperature (LM-35DZ) and relays	WN-USB ZigBee module
[20]	32-bit ARM microcontroller	X10, Serial, EIB, ZigBee, Bluetooth, DTMF, CAN and GSM/GPRS/UMTS	Star	Multiple I/O pins for attaching sensors and actuators	Proprietary board based on a 32-bit ARM microcontroller
[21]	Android tablet and Arduino MEGA with an Ethernet shield	X10, ZigBee	Tree	Light and switch modules	Arduino
[22]	32-bit ARM-Cortex M3 microcontroller	ZigBee	Tree	The paper only suggests different sensors and actuators for the HAS, but it is actually not implemented	CC2530
[23]	-	EIB/KNX, WiFi, Bluetooth and ZigBee	Star	Environmental (door/window opening, light, temperature), biometric (wrist pulse oximeter, body scale, wrist blood pressure, ear thermometer) and actimetry (movement detection, bed/chairs presence, lighting control, water and electricity meter) sensors and actuators	WaspMote platform
[24]	Raspberry Pi 2	WiFi, ZigBee, IrDA, Ethernet	Star	Smart plugs, IP cameras	Raspberry Pi 2 (the controller also acts as sensor node)
[25]	Cubietrack board (ARM-Cortex A7)	WiFi, ZigBee	Star	Temperature, light and current sensors (ACS712). Relays and dimmers.	ESP8266, Xbee
[41]	PC	ZigBee	Star	Smart plugs	ATmega328P microcontroller
[43]	-	IEEE 802.15.4	Tree	-	MICAz motes
[44]	-	WiFi, IR	Star	Dust sensor	Commercial UART-WiFi module
[45]	Raspberry Pi 2 model B	WiFi	Star	-	ESP8266
[46]	PC	WiFi	Star	Luminosity sensor (LDR), LED and buzzer	ESP8266

**Table 4:** Comparison of the features of commercial home automation systems.

Solution	HomeSeer	Qivicon	Loxone	Domintell
Protocols	Insteon, UPB, Wi-Fi, X10, PLC-BUS, Modbus, Z-Wave	Wi-Fi, ZigBee	KNX, DMX, Modbus, RS232, RS485, EnOcean, Loxone Air	S-Bus
Transmission	Wired and wireless	Wireless	Wired and wireless	Wired
Locking system	Yes	Yes	Yes	Yes
Temperature	Yes	Yes	Yes	Yes
Media center	Yes	Yes	Yes	Yes
Lighting	Yes	Yes	Yes	Yes
Environmental control	Yes	Yes	Yes	Yes
Video surveillance	Yes	Yes	No	No
User experience	Acceptable	Acceptable	Good	Good
Variety of peripherals	High	Very high	Medium	Medium
Technical security	Yes	Yes	Yes	Yes
Anti-intrusion	Yes	Yes	Yes	Yes
System Requirements	800 MHz Quad-Core CPU, 1 GB RAM-1.5 GHz Dual-Core CPU, 2 GB RAM-1.8 GHz Dual-Core CPU, 2 GB RAM	1-Core ARM v11, 600 MHz, 512 MB RAM	400 MHz, 64 MB RAM	Not provided by the manufacturer
Price (€)	1000–1200	1300	1250	900

**Table 5:** Feature comparison of the most relevant open-source home automation software.

Software/Feature	Main Task	License	Main Development Language	Web Interface	Protocols	Low-Cost Gateway Support	Messaging Service	API	Plugins	Documentation
Ago Control [95]	HAS	GPL v3	C++	Yes	Many	Yes	AMQP	No	A few	Good
						(e.g., Raspberry Pi or PogoPlug)	(MQTT supported)	(but JSON-RPC interface)		
Calaos [96]	Control and monitor homes	GPL v3	C++	Yes	A few	Yes	-	Yes	Under development	Limited
					(under development)	(e.g., Raspberry Pi, Cubieboard)		(JSON-based)		(partly in French)
Domticz [97]	HAS	GPL v3	C++	Yes	Many	Yes	MQTT	Yes	Many	Extensive
						(e.g., Raspberry Pi or FreeNAS)			(JSON-based)	
Fhem [98]	HAS	GPL v2	Perl	Yes	Many	Yes	MQTT	Yes	Many	Extensive
						(e.g., Raspberry Pi, NAS)		(ASCII commands)		(partly in German)
FreeDomotic [99]	IoT framework	GPL v2	Java	Yes	A few	Yes	MQTT	Yes	Many	Extensive
						(e.g., Raspberry Pi)		(REST API, under development)		(partly in Italian)
Home-Assistant [100]	HAS	Apache 2.0	Python 3	Yes	Many	Yes	MQTT	Yes	Many	Extensive
						(e.g., Raspberry Pi 3)		(REST/Python/Websocket APIs)		
Home Genie [101]	HAS	GPL v3	Javascript / C# / Python / Ruby	Yes	Many	Yes	MQTT	Yes	Many	Extensive
						(e.g., Raspberry Pi, CubieTrack)		(REST API and SDK)		
ioBroker [102]	IoT platform	MIT	Javascript / Node.js	Yes	Many	Yes	MQTT	Yes	Many	Extensive
						(e.g., ARM-based boards)		(REST API)		
Jeedom [103]	HAS	GPL v2	PHP	Yes	Many	Yes	MQTT	Yes	Many	Extensive
						(e.g., Raspberry Pi 2 or 3, Synology NAS)		(JSON RPC and HTTP-based)		(partly in French)
LinuxMCE [104]	Home automation suite	GPL/Pluto	C / C++	No	Many	Yes	-	No	Many	Extensive
				(only for administration)		(e.g., Raspberry Pi)				
MajorDoMo [105]	HAS	MIT	PHP	Yes	Many	Yes	MQTT	Yes	Many	Extensive
						(e.g., Raspberry Pi 2 or 3)		(HTTP-based)	(Addons market)	(partly in Russian)
MyController [106]	Sensor controller	Apache 2.0	Java	Yes	Many	Yes	MQTT	Yes	Many	Extensive
						(e.g., Raspberry Pi)		(REST)		
OpenHAB [107]	HAS	EPL v1	Java	Yes	Many	Yes	MQTT	Yes	Many	Extensive
						(e.g., ARM-based boards)		(REST)		
OpenNetHome [108]	HAS	GPL v3	Java	Yes	Many	Yes	MQTT	Yes	Many	Extensive
						(e.g., Raspberry Pi)	XMPP	(REST)		
Pimatic [109]	Home automation framework	GPL v2	Node.js	Yes	Many	Yes	MQTT	Yes	Many	Extensive
						(e.g., Raspberry Pi)	XMPP	(HTTP-based)		

**Table 6:** Main characteristics of ESP8266-based boards.

Model	ESP-01	ESP-12	ESP-201	NodeMCU v1.0	Sparkfun Thing	Adafruit Huzzah	WeMos D1 Mini
ESP Version	ESP-01	ESP-12	ESP-201	ESP-12E	ESP-12E	ESP-12E	ESP-12E
Number of GPIO pins	2	11	11	11	11	11	11
Memory	512 KB	512 KB	512 KB	4 MB	4 MB	4 MB	4 MB
Ease of integration in prototypes	Medium	No	High	High	High	High	High
Power voltage	3.3 V	3.3 V	3.3 V	3.3 V–6 V	3.3 V–6 V	3.3 V–6 V	3.3 V–6 V
Form factor	Small	Medium	Large	Large	Large	Medium	Small
Price	$3	$3	$3	$6.5	$16	$10	$4
Compatible with Arduino IDE	Yes	Yes	Yes	Yes	Yes	Yes	Yes
Serial comms.	It needs a USB adapter	It needs a USB adapter	It needs a USB adapter	It needs a USB adapter	micro-USB	It needs a USB adapter	micro-USB

**Table 7:** Sensors used by ZiWi’s nodes.

Sensor	Identifier	Output	Operation Range	Precision	Input Voltage Range	Consumption	Price
Temperature	LM35	Analog	−55, +150 C	±0.5 C	4–30 V	114 µA	$3
Temperature	TMP36	Analog	−40, +125 C	±1–2 C	2.7–5.5 V	40 µAA	$1.50
Humidity	HIH-5030	Analog	0–100%	±3%	2.7–5.5 V	200 µA	$10
Luminosity	LDR	Analog	1–1000 lx	-	max. 100 V	max. 75 mA	$0.5
Temperature and Humidity	DHT11	PWM	0–50 C / 20–80%	± 2 C / ± 5%	3–5 V	200 µA	$5
Motion	Parallax PIR sensor rev. A	Digital	0–6 m	-	3–5 V	100 µAA	$10
Current	ACS712	Analog	Up to 30 A	±1.5%	4.5–5.5 V	10 mA	$3–$5

**Table 8:** Response time comparison of ZiWi with other state-of-the-art systems.

System	Scenario	Response Time
ZiWi-Fog	Turn on a light when low luminosity is detected	20 ms
ZiWi-Fog	Switch on a relay	18 ms
ZiWi-Fog	Switch off a relay	16 ms
ZiWi-Cloud	Detect and notify alert through Telegram	2.735 s
ZiWi-Cloud	Turn on a light when low luminosity is detected through IFTTT	51.388 s
[19]-ZigBee	Switch on/off relay	2 s
[19]-GSM	Switch on/off relay	25 s
[25]	Read sensor value	0.5 s
[25]	Act on actuator	0.5 s
[41]	Detect and react to a shortcut	1233.19 µs
[43]	Collect sensor values and react depending on them	32.9 ms

**Table 9:** ZigBee success delivery rate in the presence of WiFi cross-interference.

Node Distance	WiFi Enabled	Channel Overlapping	Average Local RSSI (dBm)	Average Remote RSSI (dBm)	Packets Sent	Packets Received	TX Errors	Packets Lost	Packet Delivery Success Rate (%)
Short	No	No	−36	−36	100	100	0	0	100%
Short	Yes	No	−42	−44	100	96	3	1	96%
Short	Yes	Yes	−49	−44	100	86	14	0	86%
Medium	No	No	−59	−54	100	100	0	0	100%
Medium	Yes	No	−55	−50	100	96	4	0	96%
Medium	Yes	Yes	−56	−51	101	78	20	3	77.23%

**Table 10:** Node consumption with and without encryption.

Mode	Sensor Node Consumption (mA)			Actuator Node Consumption (mA)		
	Without Encryption	With APS Encryption	Difference	Without Encryption	With SSL Encryption	Difference
Average	23.494	24.006	+2.13%	39.369	87.800	+55.16%
In Transmission	55.200	56.400	+2.13%	96.950	117.74	+17.66%
Idle	12.925	13.208	+2.14%	20.175	21.940	+8.04%

## Abbreviations

The following abbreviations are used in this manuscript:

ADC	Analog-to-Digital Converter
AMQP	Advanced Message Queuing Protocol
AP	Access Point
API	Application Programming Interface
APS	Application Support Sublayer
BLE	Bluetooth Low Energy
HAS	Home Automation System
HVAC	Heating, Ventilation and Air-Conditioning
IoT	Internet of Things
IPSP	Internet Protocol Support Profile
ISM	Industrial, Scientific and Medical
JMS	Java Messaging Service
JVM	Java Virtual Machine
MOM	Message-Oriented Middleware
MQTT	Message Queuing Telemetry Transport
MOM	Message-Oriented Middleware
MTC	Machine-Type Communications
NTP	Network Time Protocol
RPC	Remote Procedure Call
SoC	System on Chip
SMD	Surface-Mount Device
SSL	Secure Sockets Layer
STOMP	Simple (or Streaming) Text Oriented Messaging Protocol
XMPP	Extensible Messaging and Presence Protocol
VPN	Virtual Private Network
6LowPAN	IPv6 over Low-Power Wireless Personal Area Networks
